# High-resolution temporal profiling of the human gut microbiome reveals consistent and cascading alterations in response to dietary glycans

**DOI:** 10.1186/s13073-020-00758-x

**Published:** 2020-07-03

**Authors:** Richard Creswell, Jie Tan, Jonathan W. Leff, Brandon Brooks, Michael A. Mahowald, Ruth Thieroff-Ekerdt, Georg K. Gerber

**Affiliations:** 1 Department of Pathology, Brigham and Women’s Hospital, 60 Fenwood Road, Boston, MA 02115 USA; 2 Kaleido Biosciences, Lexington, MA 02421 USA; 3 Present Address: Prescient Metabiomics, Carlsbad, CA 92008 USA; 4 Present Address: LEO Pharma A/S, Ballerup, Denmark; 5 Present Address: Sojournix Inc., 400 Tottenpond Rd, Waltham, MA 02451 USA; 6 Harvard Medical School, Boston, MA 02115 USA

**Keywords:** Microbiome, Dynamics, Glycans, Human, Gastrointestinal, Time-series

## Abstract

**Background:**

Dietary glycans, widely used as food ingredients and not directly digested by humans, are of intense interest for their beneficial roles in human health through shaping the microbiome. Characterizing the consistency and temporal responses of the gut microbiome to glycans is critical for rationally developing and deploying these compounds as therapeutics.

**Methods:**

We investigated the effect of two chemically distinct glycans (fructooligosaccharides and polydextrose) through three clinical studies conducted with 80 healthy volunteers. Stool samples, collected at dense temporal resolution (~ 4 times per week over 10 weeks) and analyzed using shotgun metagenomic sequencing, enabled detailed characterization of participants’ microbiomes. For analyzing the microbiome time-series data, we developed MC-TIMME2 (Microbial Counts Trajectories Infinite Mixture Model Engine 2.0), a purpose-built computational tool based on nonparametric Bayesian methods that infer temporal patterns induced by perturbations and groups of microbes sharing these patterns.

**Results:**

Overall microbiome structure as well as individual taxa showed rapid, consistent, and durable alterations across participants, regardless of compound dose or the order in which glycans were consumed. Significant changes also occurred in the abundances of microbial carbohydrate utilization genes in response to polydextrose, but not in response to fructooligosaccharides. Using MC-TIMME2, we produced detailed, high-resolution temporal maps of the microbiota in response to glycans within and across microbiomes.

**Conclusions:**

Our findings indicate that dietary glycans cause reproducible, dynamic, and differential alterations to the community structure of the human microbiome.

## Background

Dietary glycans are known to alter the growth and activity of microbes in the human gut, and certain dietary glycans have been shown to have a beneficial effect on health without being directly digested by the host [1, 2]. While beneficial health outcomes have been linked to a wide range of glycan compounds, the mechanisms through which they affect the gut microbiome and how this leads to an alteration of host physiology remains unclear. Moreover, how the microbiome changes in composition and function with glycan administration, and the consistency and temporal patterns of these responses, remains poorly understood. Characterizing these responses to different compounds and across individuals, with frequently sampled timepoints, is thus an important priority for microbiome research to further understanding of diet-induced responses [3]. Such studies have the potential to provide predictive insights into how glycans and other dietary compounds can be used to improve health or treat disease.

Previous work has shown shifts in the composition of the microbiome with individual dietary glycans in small clinical studies [4]. Generally, these studies either focus on certain bacterial taxa, a small number of timepoints, or a single dose and often only include a limited assessment of the variability in response across participants. Nonetheless, these studies have been instrumental in demonstrating the potential for dietary glycans to drive meaningful shifts in the microbiome and have provided evidence that these shifts are linked to functional outcomes (e.g., [5]).

The human gut microbiome is inherently temporally dynamic, due to various factors including dietary intake [6, 7]. Metabolism of dietary glycan compounds in the gut can be a complex process mediated by many different organisms that can interact synergistically over time [8]. For example, bacterial cross-feeding interactions have been shown to occur in the gut, whereby one bacterial species performs primary degradation of polysaccharides and then another bacterial species grows abundantly on the resulting secondary products [9]. Characterizing the dynamic responses of the microbiome to glycans has direct relevance to the effective use of these compounds to improve human health. Necessary factors to understand include the onset, duration, and durability of their effects on the microbiome.

To characterize temporal dynamic responses of the microbiome to glycans in detail, we conducted a set of temporally dense and high-taxonomic resolution studies. This investigation was comprised of three studies of consuming two different glycans (fructooligosaccharides [FOS] and polydextrose [PDX]). Participants’ microbiome compositions from fecal samples were profiled using shotgun metagenomic sequencing, with multiple samples collected before, during, and after the intake of each glycan (~ 4 samples/week) over the course of 10 weeks (Additional file 1: Figure S1). We assessed the impact of glycan dose on responses by delivering different doses of compounds to cohorts. We additionally performed a crossover study between FOS and PDX (Additional file 1: Figure S1) to investigate the effect of prior glycan consumption on microbiome responses.

The two glycans we investigated differ substantially in their chemical structures and are thus likely to result in different response patterns in the microbiome. FOS, a mixture of fructose units linked by beta-1,2-bond and a degree of polymerization (DP) between 2 and 8, was one of the earliest described nondigestible food ingredients [1] and is widely used in food products including infant formulas due to advantageous chemical properties and potential benefits to health [10]. It is fermented in the colon [11], and human studies have shown FOS to increase *Bifidobacterium* growth [12–14]. In contrast, PDX is a mixture of nondigestible polysaccharides comprised of varying lengths of glucose monomers linked with diverse glycosidic bonds and small amounts of sorbitol and citric acid with an average DP of 12 [15, 16]. In human participants, administration of PDX has been shown to alter the composition of the gut microbiome by increasing the *Bacteroides* to *Firmicutes* ratio and shifting the proportions of specific taxa, including decreases in *Eubacterium*, *Roseburia*, *Ruminococcus*, *Dorea*, and *Lachnospiraceae* and increases in *Parabacteroides* [5, 17]. PDX has been shown to have physiological effects on the host including a reduction in appetite, shift in lipid metabolism, and an improvement in immune function [15].

To analyze the rich time-series data collected from our studies, we developed MC-TIMME2, an improved version of our earlier Microbial Counts Trajectories Infinite Mixture Model Engine (MC-TIMME [18]), which simultaneously infers temporal patterns induced by perturbations and groups of microbes sharing these patterns from microbiome data, using a nonparametric Bayesian technique [19, 20]. MC-TIMME has been successfully applied to gain biological insights into the responses of the microbiome over time to antibiotics [18], infection [21], and diet [22]. In contrast to other techniques for microbiome time-series analysis (e.g., [23–25]), MC-TIMME detects differences *within* a single participant’s microbiome, enabling personalized response characterization. To address the dense time-series data collected and our interventional experimental design, we made several key extensions to the original MC-TIMME model and algorithm, including: (a) stochastic dynamics, to model unmeasured sources of temporal variation that occur in diverse human microbiomes; (b) an explicit model of the perturbing substance, to account for pharmacokinetics of the compound and differing doses administered; (c) a tailored measurement error model for metagenomics sequencing data including nonparametric overdispersion; and (d) a multi-level clustering model, to allow for characterization of shared growth kinetic properties due to phylogenic relationships and shared responses to the compound that may be due to common metabolic capabilities in phylogenetically distant bacteria.

We analyzed the data from our studies using both standard statistical/ecological approaches and MC-TIMME2. Overall, we observed consistent effects of the glycans across individuals, in terms of changes in the composition of the microbiota and specific taxa, and distinct effects between structurally dissimilar glycans. Further, we saw significant changes in abundances of microbial genes that encode carbohydrate-active enzymes. Detailed temporal modeling of these responses reveals consortia of bacteria that respond at different rates and with distinct trajectory patterns, highlighting complex but consistent temporal responses to glycans.

## Methods

### Study design

Three clinical studies were conducted to assess the effects of fructooligosaccharides (FOS) and polydextrose (PDX) on the gut microbiome of healthy male and female human participants age 18–45 years. FOS used in the studies is a brand of FOS called Orafti® P95 dry powder manufactured by BENEO. PDX used in the studies is a brand called Litesse® Ultra™ manufactured by Danisco. The first two studies (K001 and K002) had similar designs except that K001 used FOS as the study product, and K002 used PDX as the study product (Additional file 1: Figure S1). Both were randomized, open-label studies that included three cohorts, which received different feeding amounts of the study products. Following a 2-week baseline/run-in period, participants were randomized via computer-generated codes on study day 0 to one of the three cohorts. Participants consumed the study products during two feeding periods of 2 weeks each (feeding1 and feeding2). There was a 4-week washout period following the feeding periods during which participants were monitored but no study product was consumed. In K001, participants in cohort 1 were instructed to consume 2.5 g BID of FOS per day during feeding1 and feeding2. In cohort 2, 2.5 g BID during feeding1 and 5 g BID during feeding2. In cohort 3, 5 g BID during feeding1 and 10 g BID during feeding2. In K002, participants in cohort 1 were instructed to consume 7.5 g BID of PDX per day during feeding1 and feeding2. In cohort 2, 10 g BID during feeding1 and 20 g BID during feeding2. In cohort 3, 20 g BID during feeding1 and 30 g BID during feeding2. The study products were consumed twice daily, dissolved in water. The study product amounts were comparatively greater in K002 as compared to K001 due to reported higher tolerability for PDX than FOS [16, 26]. A third randomized, double-blinded crossover study (K003) was conducted to assess whether effects on the microbiome were reproducible and/or dependent on the order of dosing. Following a 2-week run-in period, healthy participants were randomized into one of two cohorts on study day 0. Participants in cohort 1 received 5 g BID of FOS during the first 2-week feeding period (feeding1), discontinued consumption of FOS during the subsequent 4-week period (washout1), received 25 g BID of PDX during the second 2-week feeding period (feeding2), and were followed for a second 4-week period (washout2) after discontinuation of the consumption of PDX. Cohort 2 followed an identical plan except that participants in the cohort received the study products in the reverse order across the two feeding periods. Study products were consumed as in the two earlier studies. The differences between the intended and actual consumption amount are summarized in Additional file 1: Figure S2. Participants were instructed to maintain their normal diet throughout the study. For each study, we randomized 10 participants per cohort. The demographic information of participants is listed in Additional file 1: Table S1. Participants with a major protocol deviation or completely missing data from one of the study periods were excluded from downstream analyses.

### Sample collection and sequencing

Fecal samples were collected in order to characterize the gut microbiome of the participants. Participants were provided with fecal sample collection instructions. Briefly, participants were instructed to place a stool collection bowl and holder under toilet seat in the center rear of the toilet and lower seat, to ensure that the collection unit was securely in place. Participants then completed bowel movement on the toilet, into the sample collection unit. To generate the swab sample, participants opened the red capped swab collection tube (BD BBL CultureSwab EZ II single head foam swab), stuck swab into the center of the stool to heavily coat swab head, returned swab back into the collection tube, and twisted cap back onto collection tube to seal completely. Swab samples were then placed immediately in the freezer or were temporarily placed in the provided insulated cooler bag with four frozen freezer packs until participants could access their freezer. To bring collected samples back to the clinical site, participants placed fecal swabs in the provided insulated cooler containing the freezer packs.

Fecal microbiomes were characterized using a shotgun DNA sequencing-based approach similar to one previously described [27]. Briefly, fecal swabs were removed from the collection device quickly and cut into Qiagen’s DNeasy PowerSoil extraction plates. Extraction plates remained on dry ice until all fecal swabs were transferred. Plates were stored at – 80 °C until analysis. Extraction plates were shipped on dry ice to CoreBiome, Inc. for downstream extraction and library preparation. Extracts were quantified using the Quant-iT PicoGreen dsDNA assay (Thermo Fisher). Libraries were prepared using the NexteraXT kit and a HiSeq 1 × 150-cycle v3 kit (Illumina) was used to sequence samples. The taxa count tables from raw sequencing data were generated using the SHOGUN pipeline [27] and have been made publicly available (see the “Availability of data and materials” section). The database used here was generated by selecting up to the first 20 strains per species in RefSeq v87 by first choosing genomes with assembly level annotated as “Complete Genome,” then “Chromosome,” then “Scaffold,” and then “Contig.” All calculations and visualizations were conducted on an operational taxonomic unit (OTU) table that excluded OTUs that could not be resolved to the species level. This table was rarefied to 10,000 without replacement and used for downstream analyses.

### Microbiome data analysis

We assessed the effects of FOS or PDX on microbiome diversity by comparing median alpha (Shannon) diversity values between each of the feeding periods and the baseline/run-in period and testing the significance of differences via paired Wilcoxon signed-rank tests. *P* values were false discovery rate (FDR) corrected for multiple comparisons across periods.

To assess the overall effect of FOS or PDX on microbiome community composition, we first calculated Bray-Curtis dissimilarities between every sample pair within each participant based on the square-root transformed relative abundances of the bacterial species. We determined whether the compositions during the feeding periods differed from the baseline/run-in period using permutational analysis of variance (PERMANOVA) as implemented by the “adonis” function in the vegan package [28]. Participant identity was included for the “strata” parameter to limit permutations to within participants. We next assessed FOS or PDX’s effect on community dissimilarity. We tested the differences between the dissimilarities between one subject’s every sample from baseline/run-in and every sample from another period and the dissimilarities within samples from baseline/run-in using Kruskal-Wallis test followed by Dunn’s post hoc test (as implemented in the dunn.test R package [29] with Benjamini-Hochberg correction).

To evaluate whether the high-dose cohort induced higher community shift than the lower dose cohorts, we again looked at the pair-wise dissimilarity between every baseline/run-in sample and every feeding-period sample across three cohorts and tested their differences between cohorts using a Kruskal-Wallis test followed by Dunn’s post hoc test with Benjamini-Hochberg correction.

We assessed whether individual taxa significantly differed between feeding periods and the run-in period using a linear mixed effect model as in [30]. Specifically, for each taxon, we rank transformed its relative abundance data and fit a linear mixed effect model (using the lme function from nlme R package [31]) with period as a fixed effect and participant as a random effect. The test results for all taxa were corrected using FDR. Only taxa with relative abundances greater than or equal to 0.1% in either the run-in or feeding periods were examined. All aforementioned analyses were performed using R version 3.5.3 [32].

### Differential abundance analysis of genes encoding CAZymes

We obtained functional information for each species using a custom annotation pipeline derived from [33]. For each species under analysis, all genomes in NCBI corresponding to that species were downloaded. We used the dbCAN database for CAZymes [34, 35]. The annotation pipeline was applied to the genomes, resulting in a map from strains to CAZymes. A map from species to functional units was created by merging the results for all strains within each species. For each species, we thus obtained a set of CAZymes. For each CAZyme, the abundances of species that passed filtering criteria (see the “Initial filtering of taxa” section below) in each participant and annotated as having the gene were aggregated together and log2 fold changes (baseline versus feeding period) were calculated. Aggregated fold changes were similarly calculated for taxa not annotated to have the gene. These fold changes were then compared using the two-sided Wilcoxon rank-sum test (with *p* values adjusted for multiple hypothesis testing using the Benjamini-Hochberg method and FDR < 0.01.) To be precise: Consider a CAZyme *c*. For each subject *s*, let *A*^*s*,*i*^*, i* = 1,...*N*_*s*_ denote the series of *N*_*s*_ timepoints of aggregated relative abundance of all species in subject *s* that are annotated as having CAZyme *c*, and let *B*^*s*,*i*^ similarly denote the time series of aggregated relative abundance of all species in *s* not annotated to have CAZyme *c*. Fold changes for taxa annotated as having the gene are calculated according to the following:

*FC*_*A*_(*s*) = median(*A*^*s*,*i*^ for *i* in the feeding periods)/median(*A*^*s*,*i*^ for *i* in the baseline periods).

*FC*_*B*_(*s*) is calculated analogously. The Wilcoxon test is then performed on the set of log2 *FC*_*A*_(*s*) versus the set of log2 *FC*_*B*_(*s*).

### MC-TIMME2 model and software

#### Software

MC-TIMME2 was implemented in Python 3 using the Scipy ecosystem and Numba [36–39]. The software and source code to reproduce analyses in the manuscript is publicly available under the GNU General Public License [40]. Configuration settings for the model and inference are specified in a text-based configuration file, and the software is run from the command line. Data is loaded from three files: an OTU table containing the counts for each taxon in each sample, a Sample Info file containing the time and participant of each sample, and a Treatment Info file containing the time and quantity of each administered dose of the compound. Additionally, a phylogenetic tree for the taxa being analyzed must be provided. In order to learn the parameters of the sequencing measurement noise model, replicate data must be provided. This consists of another OTU table containing the count data for a set of technical replicates. Here we used 10 technical replicates of shotgun sequencing from a single human stool sample. DNA was extracted using Qiagen’s PowerFecal kit and DNA quantification, library preparation, and sequencing were done at CoreBiome, Inc. as described earlier. Inference for the parameters of the measurement noise model was performed upfront and separately from the rest of the model.

As the software performs posterior inference, it produces an output file containing the posterior samples for all parameters in HDF5 format. Once inference is complete, the software includes functionality to generate output files from this file, including visualizations for each time-series, reconstructed perturbation groups, and text-based tables showing the carrying capacities, pharmacokinetic parameters, onset times, and cluster memberships for each time-series.

#### Initial filtering of taxa

Before running the model, we apply two initial filters to remove very low abundance taxa trajectories. The first filter removes any species which does not achieve a mean abundance (across the trajectory) of at least 0.0005 in at least 25% of the participants. The next filter is applied to each individual trajectory and removes any trajectory that does not achieve at least 15 counts in any three consecutive timepoints.

#### Model details

##### Double Dirichlet process clustering

Two levels of Dirichlet process mixture model clustering are employed. Individual species-participant time-series are probabilistically assigned in the model to microbe groups, with group assignments denoted by *z*_*so*_ for species *o* in participant *s*. Each microbe group *k* is itself assigned either to a null perturbation effect or to a perturbation group in the top level of the double Dirichlet process. Let *e*_*k*_ denote the assignment to a perturbation group for microbe group *k*. Phylogenetic information is incorporated into clustering using a potential function *ψ*:

1 $$ \psi \left(o,{\lambda}_k\right)=\exp \left(-{\zeta}_0d\left(o,{\lambda}_k\right)+{\zeta}_1\right) $$

Each microbe group probabilistically selects a representative member *λ*_*k*_. Then, the likelihood for each time-series being assigned to the microbe group *k* incorporates the phylogenetic distance between that species and the representative. Letting *Ω*_*m*_ indicate the perturbation parameters for perturbation cluster *m*, we write the probability of assigning species *o* in participant *s* as follows:

2 $$ P\left({z}_{so}=k|{x}_{so},{e}_k,{\lambda}_k\right)\propto P\left({z}_{so}=k\right)P\left({x}_{so}|{\Omega}_{e_k}\right)\ \psi \left(o,{\lambda}_k\right) $$

##### Pharmacokinetic model

The inputs to the model include the participant-specific doses *d*_*si*_ and their times of administration *a*_*si*_. The level of the compound over time, *c*_*s*_, is modeled using first-order pharmacokinetics, with a participant-specific elimination rate *κ*_*s*_.

3 $$ {c}_s(t)={\sum}_i{d}_{si}\exp \left(-{\kappa}_s\left(t-{a}_{si}\right)\right)I\left(t>{a}_{si}\right) $$

##### Dynamical model

We use a stochastic logistic growth model for baseline microbial dynamics. In the absence of a perturbation, each species is assumed to follow a stochastic logistic growth model with a growth rate *α*, a self-limiting term *β*, and a process variance *σ*_*so*_^*2*^ specific to each time-series *so*.

4 $$ {\mu}_{so}\left({t}_{s,i}\right)={x}_{so}\left({t}_{s,i-1}\right)+{\alpha}_{so}{x}_{so}\left({t}_{s,i-1}\right){\Delta}_{s,i}+{\beta}_{so}{x}_{so}{\left({t}_{s,i-1}\right)}^2{\Delta}_{s,i} $$

5 $$ {x}_{so}\left({t}_{s,i}\right)\sim N\left({\mu}_{so}\left({t}_{s,i}\right),{\Delta}_{s,i}{\sigma}_{so}^2\left({t}_{s,i}\right)\right) $$

The process variance takes different values on and off the perturbation period, allowing the model to capture changes in variance which may accompany the administration of the compound.

6 $$ {\sigma}_{so}^2(t)=\left\{\begin{array}{c}{c}_{so}\kern1.25em \mathrm{t}\ \mathrm{off}\ \mathrm{perturbation}\\ {}{\hat{c}}_{so}\kern1.5em \mathrm{t}\ \mathrm{on}\ \mathrm{perturbation}\end{array}\right. $$

##### Auxiliary trajectory

Inference efficiency depends on the use of a normal distribution (not truncated) in the stochastic dynamical Eq. (5). However, it is desirable to avoid negative values for the trajectory *x*, which are physically unrealistic and unstable. This is achieved using a relaxation method which we previously developed [41]. This method introduces a strictly positive auxiliary trajectory which is closely coupled to the dynamical trajectory. The effect of this coupling is to tend to keep *x* away from negative values without forcing a computationally intractable hard constraint.

##### Model of dose-dependent perturbation

The baseline growth rate *α*_*so*_ in (4) is modulated by a perturbation as follows:

7 $$ {\alpha}_{so}^{\prime}\left({t}_{s,i}\right)={\alpha}_{so}\ \left(1+{\gamma}_{1m}{w}_{sm}\left({t}_{s,i}\right)h\left({c}_s\left({t}_{s,i}\right);{r}_m\right)+{b}_{so}{\gamma}_{2 so}{p}_{so}\left({t}_{s,i}\right)\right) $$

In (7), *w* is a step function specifying when the perturbation is active; *w* is defined by an on-time in days and a duration relative to the dose administration period. Here, *h* is a sigmoidal transfer function which reshapes the participant-specific pharmacokinetic trajectories *c*_*s*_ into a perturbation magnitude over time.

8 $$ h\left({c}_s(t);{r}_m\right)=\frac{2}{1+\exp \left(-{r}_m{c}_s(t)\right)}-1 $$

Each perturbation cluster also has the parameter *γ*_*1m*_ controlling the strength of the perturbation.

The parameters *b*_*so*_, *γ*_*2so*_, and *p*_*so*_ allow for a persistent effect in which the growth rate continues to be modulated even after the compound is excreted. Occurrences of persistent effects are probabilistically selected to be on or off, and when they are selected to be on, the parameters are specific to an individual time-series. Here, *b*_*so*_ is the indicator variable that probabilistically selects whether this effect is on, with the prior on *b*_*so*_ favoring no persistence. Here, *γ*_*2so*_ is the magnitude of the persistent effect and *p*_*so*_ is a step function that turns on at the last dose administration time and continues for *t*_*so*_^persist^ days.

##### Measurement model

The observed data are sequencing counts *y*. The counts are assumed to be drawn from a negative binomial distribution, which has been used extensively to model microbiome sequencing counts data [42]. In order to model the noise properties of the shallow shotgun sequencing data used in these studies, we used a mixture model for the replicates data (separate from the main model of microbiome responses). The replicates data consists of counts *y*_*io*_ for species *o* in replicate *i*, with each species assumed to have one latent mean relative abundance μ_o_ across all replicates. Thus, the sample-specific mean *η*_*io*_ equals *ν*_*i*_ multiplied by *μ*_*o*_. For the overdispersion, we use a Dirichlet process mixture model of negative binomial components, where each component has a single overdispersion parameter. Therefore, conditional on indicator variables *z*_*o*_,

9 $$ {y}_{io}\mid {z}_o\sim NB\left({\eta}_{io},{\epsilon}_{z_o}\right) $$

Inference is performed for this model, resulting in a consensus set of negative binomial components, each with an overdispersion value and a mixture weight. To use this result in the main model, assignments to the consensus clusters are marginalized out:

10 $$ {y}_{so}\left({t}_s\right)\sim {\sum}_j{\pi}_j^{\mathrm{noise}} NB\left({\eta}_{so}\left({t}_s\right),{\epsilon}_j\right) $$

### Phylogenetic tree

MC-TIMME2 takes as input a phylogenetic tree between all species in the model. For each species in the database used for taxonomic calling described earlier, the aligned 16S rRNA sequences for all strains corresponding to that species were selected from RDP [43]. Several species in the database were not present in RDP. For those species, unaligned 16S rRNA sequences were obtained from NCBI RefSeq [44] and aligned to the RDP alignment using QIIME/PyNAST [45]. Using this alignment of 16S rRNA sequences, FastTree was used to generate a phylogenetic tree [46].

### Comparison of onset times

For comparison of onset times, we restricted analyses to time-series with strong evidence of response (Bayes factor > 100). Additionally, for clarity, we tested only pairs of bacterial families if each family had at least 10 responding time-series. We used the Mann-Whitney test to compare the two sets of posterior median onset times. *P* values are corrected for multiple comparisons using the Benjamini-Hochberg procedure.

### Consensus clustering

To obtain a single consensus clustering from the MCMC samples, we used an agglomerative clustering method similar to that used in [47]. Reconstruction was applied to those time-series for which there was strong evidence of a response (Bayes factor > 100). First, the microbe co-clustering frequencies were used to form an affinity matrix between each time-series. Agglomerative clustering up to the mode of the number of microbe groups was performed to create a set of consensus microbe groups. To form consensus perturbation groups, an affinity matrix between the consensus microbe groups is formed, with the affinity between each pair of consensus microbe groups given by the average of all pairwise perturbation co-clustering frequencies among the members of the consensus microbe groups.

### Visualization and filtering of perturbation groups

For visualization, we applied post hoc filtering to the consensus perturbation groups. First, we filtered species, keeping only those species which appeared in any perturbation group at least 6 times (corresponding to a response in 25% (FOS) or 23% (PDX) of participants). Next, we filtered out perturbation groups which did not have among their members at least 15% of participants represented. This filtered set of perturbation groups was used for visualization (Fig. 4). For functional enrichment analysis (Additional file 1: Figure S6), all species that were originally in the group were used, including those species that were filtered out in the first step for visualization. In Fig. 4, we applied an ordering to the phylogenetic tree that tends to place the more frequently responding species near the top. Each species in the tree was associated with an importance score given by the number of times a time-series of that species appeared in any perturbation group. Each slot in the grid was numbered in reverse order (the bottom slot has value 1, the second-to-bottom slot has value 2, and so on). We then maximized the dot product of the vector of species importance scores and the vector of slot values over valid configurations of the phylogenetic tree using Monte-Carlo optimization.

### Model inference, full mathematical description, and sensitivity analyses

MC-TIMME2 performs approximate posterior inference using a custom Markov chain Monte Carlo (MCMC) method. Full mathematical details of the inference algorithm and model, as well as descriptions of sensitivity analyses of hyperparameters, are given in Additional file 2: Supplemental Methods.

## Results

### FOS and PDX induce common structural changes in microbiomes despite dissimilar baseline microbiomes

We expected to see the strongest and most consistent responses in our high-dose cohorts (cohort 3, *n* = 8 for both FOS and PDX), and thus present analyses of these data first. Using standard static microbiome analysis methods, we examined baseline microbiomes of the study participants during the run-in periods and found strong cross-participant variability in diversity and composition. Alpha (Shannon) diversity scores showed 28% variation in the high-dose FOS cohort and 32% variation in the high-dose PDX cohort (Additional Figure 1: Figure S2A). Baseline abundances of the two dominant gut phyla, *Bacteroidetes* and *Firmicutes*, varied across participants, with *Bacteroidetes* ranging from 4.8 to 36% for FOS and 0.5 to 32% for PDX cohorts, and *Firmicutes* ranging from 58 to 89% for FOS and 60 to 97% for PDX cohorts (Additional Figure 1: Figure S2B). Participants shared a median of 40% species in the FOS high-dose cohort and 36% in the PDX high-dose cohort. These ranges of microbiota diversities and shared species are consistent with prior reports and similar to the magnitude of variations observed across broader Western populations [48].

**Fig. 1:**
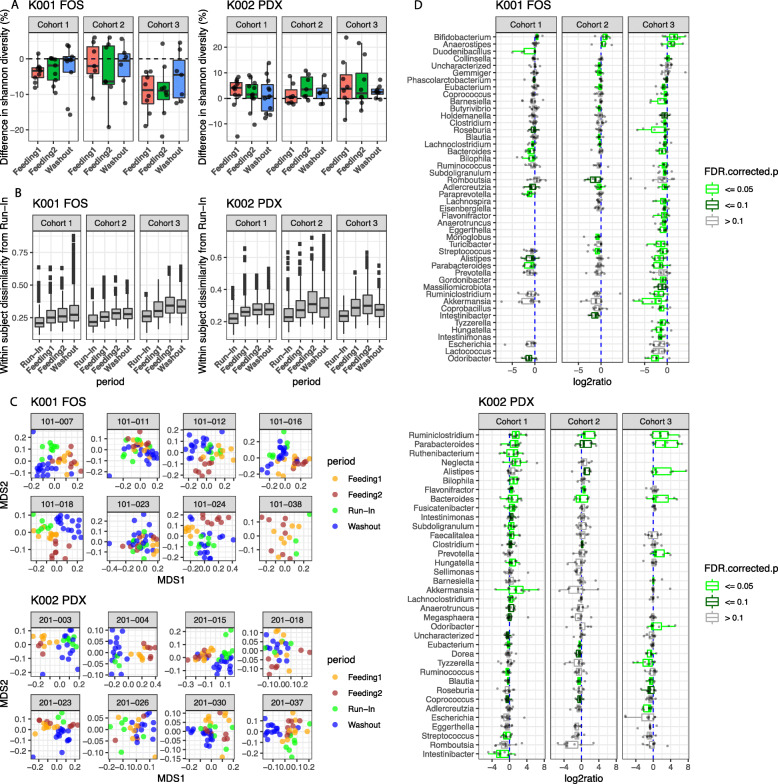
Effects of FOS and PDX on the human gut microbiome. Cohorts 3 for FOS and PDX received the highest doses of the compounds, cohorts 2 intermediate doses, and cohorts 1 the lowest doses. **a** Percent changes in alpha (Shannon) diversity between baseline and later periods (feeding1, feeding2, and washout) in each cohort receiving FOS or PDX. **b** Within-participant Bray Curtis dissimilarity from baseline samples in each cohort receiving either FOS or PDX. **c** Multidimensional scaling analysis of each participant in the high-dose cohorts receiving either FOS or PDX. Each dot represents the microbiome as measured in a fecal sample from a participant and is colored by study period. **d** Genera differentially abundant between baseline and feeding periods in each cohort receiving FOS or PDX. The boxplot summarizes taxa shifts across participants and is colored by significance level

Despite these substantial differences in baseline microbiome structure, study participants exhibited similar changes in several measures of the gut microbiota within the FOS and PDX high-dose cohorts. Participants administered FOS showed significantly decreased alpha (Shannon) diversity of their microbiomes by a median of 8.8% (adjusted *p* value = 0.02, Wilcoxon signed-rank test) during the first feeding period and a median of 9.0% during the second feeding period (adjusted *p* value = 0.04) (Fig. 1a). In contrast, participants administered PDX showed modest increases in diversity (a median of 3.7% during the first feeding period and a median of 2.0% during the second feeding period). The trend of increased diversity held in 6 of the 8 participants during both feeding periods but was not statistically significant (Fig. 1a). Interestingly, for both FOS and PDX, the effects on alpha diversity maintained the same trend even after feeding, with alpha diversity 4.5% lower in the washout period than during the baseline following FOS feeding and 2.5% higher than during the baseline following PDX feeding.

Both FOS and PDX fed participants demonstrated significantly altered microbiome compositions as assessed by beta diversity (Bray-Curtis dissimilarity) analyses between baseline and feeding periods (*p* = 0.001 for FOS and PDX, PERMANOVA test on period). Within-participant Bray-Curtis dissimilarities between baseline and feeding periods were significantly greater than the within-participant Bray-Curtis dissimilarities between baseline samples (adjusted *p* value < 0.0001 for both FOS and PDX, Kruskal-Wallis test with Dunn’s post hoc test) (Fig. 1b). We note that microbiome composition tended to shift during the first feeding period and exhibited similar shifts during the second feeding period for most participants (Fig. 1c).

Overall, FOS and PDX each induced distinct taxonomic shifts across participants’ microbiomes. To characterize these shifts, we assessed the differential abundance of taxa between baseline and feeding periods using a statistical model (see the “Methods” section). FOS feeding in general led to strong increases in *Bifidobacterium* and *Anaerostipes* genera across participants, whereas PDX feeding led to increases in a more diverse set of genera including *Parabacteroides*, *Ruminiclostridum*, *Alistipes*, *Prevotella*, *Bacteroides*, and *Odoribacter* (adjusted *p* value ≤ 0.05 for all reported genera) (Fig. 1d). A large number of genera significantly decreased in relative abundance with FOS feeding whereas only *Tyzzerella*, *Blautia*, *Dorea*, and *Adlercreutzia* significantly decreased with PDX feeding. At the species level, FOS led to significant increases in 6 *Bifidobacterium* species (Additional file 1: Figure S4). In addition to these increases in *Bifidobacterium* species, FOS also led to significant increases in *Megasphaera massiliensis* and *Anaerostipes hadrus*. PDX led to significant increases in 3 *Parabacteroides* species, 7 *Bacteroides* species, 3 *Alistipes* species, *Odoribacter splanchnicus*, *Eubacterium siraeum*, and *Clostridium leptum.*

### FOS and PDX induce similar microbiome changes at lower doses

We detected similar shifts in microbiome diversity and composition in the lower dose cohorts (cohort 1 and cohort 2), but the magnitude of these shifts tended to be lower than in the high-dose cohorts (Fig. 1a, b). Since the cohorts were not significantly different at baseline (Additional file 1: Figure S3C), these differences appear to be attributable to the effects of different doses. Specifically, microbiome compositions during the feeding period had a significantly greater Bray-Curtis dissimilarity from baseline in the high-dose cohort when compared to the low-dose cohort for FOS (adjusted *p* value < 0.001, Kruskal-Wallis test with Dunn’s post hoc test) and PDX (adjusted *p* value < 0.001). However, there were still significant shifts in microbiome community structure for FOS (*p* = 0.001, PERMANOVA) and PDX (*p* = 0.001) in cohort 1, suggesting that even relatively low doses of these compounds have an impact. Notably, for FOS, no genera showed significant increases in the low-dose cohorts that did not also show significant increases in the high-dose cohorts (Fig. 1d). For PDX, *Ruminiclostridium*, *Parabacteroides*, and *Bacteroides* showed consistent responses across the three cohorts. Interestingly, we observed a moderate but significant increase of several genera only in the low-dose PDX cohort, such as *Fusicatenibacter* and *Akkermansia*. Given the compositional nature of the data, it is possible that their absolute abundance still increased in the high-dose cohort, but the major responders such as *Parabacteroides* and *Bacteroides* had expanded disproportionately.

### Microbiome responses to FOS and PDX are reproducible in independent cohorts and robust to feeding order

To further assess the robustness of FOS and PDX effects on the microbiome, we ran a crossover design study, in which each participant was given FOS and PDX in subsequent feeding periods separated by a 4-week washout period. The doses used in the crossover study were within the range of high-dose cohorts in the previous studies. Two independent cohorts received the two study products in an opposite sequence (see the “Methods” section). This study design allowed us to: (a) determine whether the specific effects of FOS and PDX observed in the previous studies were reproducible in an independent population and, (b) determine whether responses were sensitive to prior elevated consumption of a substantially different glycan.

The direction of changes in alpha (Shannon) diversity in the crossover study was consistent with the results in the single compound feeding studies. When fed as the first compound, FOS decreased alpha diversity by a median of 4.6% while PDX increased alpha diversity by a median of 1.9% (Additional file 1: Figure S5A). We did not observe any clear carryover effect on alpha diversity for either FOS or PDX, regardless of the order of compound feeding.

The specific taxonomic shifts observed in the single compound feeding study were also largely reproduced in the crossover study. Regardless of feeding order, FOS led to significant increases in *Bifidobacterium* (Additional file 1: Figure S5B) while PDX led to significant increases in *Neglecta*, *Ruminiclostridium*, *Parabacteroides*, *Subdoligranulum*, *Fusicatenibacter*, and *Ruthenibacterium* (Additional file 1: Figure S5C) when comparing feeding to baseline periods (adjusted *p* value < 0.05 for all reported genera). We did not see evidence that these effects carried over to the subsequent administration of the second compound in the feeding sequence. Interestingly, for a few taxa, we saw possible dependence on feeding order. For example, *Ruminococcus* significantly increased during the FOS feeding period only after PDX treatment and *Faecalibacterium* and *Anaerotruncus* significantly increased during the PDX feeding period only after FOS treatment.

### Glycans promote taxa with diverse carbohydrate utilization capabilities

Given the changes observed with glycan feeding in the abundances of bacterial species in gut microbiomes, we hypothesized these changes could also result in alterations in the carbohydrate utilization capabilities of the microbiomes. To investigate this hypothesis, we analyzed changes in the abundance of Carbohydrate-Active Enzymes (CAZymes) encoding genes (Additional file 1: Figure S6; Additional file 3: Table S2). For each such gene, we performed a statistical test on the difference in the distributions of fold-change (baseline versus combined feeding periods) of aggregated abundances of taxa in participants’ microbiomes with the gene and taxa without the gene (using reference genomes to make this determination.) For FOS, we found no significant changes; for PDX we found 77 CAZyme genes were significantly more abundant during the feeding period and 6 CAZyme genes were significantly less abundant. Some of the CAZyme genes showing increased abundance are clearly directly related to metabolism of PDX, such as alpha-glucanases (e.g., GH57) and dextranases (e.g., GH66.) However, many of these CAZymes are from families not known to be directly involved in microbial metabolism of PDX and include a diverse set of microbial genes including those coding for glycoside hydrolases, polysaccharide lyases, carbohydrate esterases, and carbohydrate-binding modules. These findings suggest that PDX may promote diverse shifts in carbohydrate utilization capabilities in the microbiome; it is important to note that such shifts could be caused by either direct or indirect effects, which cannot be distinguished in this study.

### MC-TIMME2 models temporal dynamics of the response of the microbiome to perturbations and automatically identifies putative consortia of similarly responding taxa

We extended our previous MC-TIMME model [18] to enable robust analysis of the rich temporal data from our studies. As with our original MC-TIMME method, the objective of our extended method, MC-TIMME2, is to simultaneously model temporal dynamics of individual microbial taxa in each participant while automatically discovering groups of microbes across participants exhibiting common responses to the compound. Several extensions were essential due to new features of our study design and data, including a tailored model for measurement noise of metagenomics data (our previous model was calibrated on 16S rRNA sequencing data) and allowance for different doses and time-varying levels of the perturbing compound (our previous model assumed single doses and on-off kinetics.) We additionally extended the model to include capabilities expected to improve interpretability and accuracy, including flexible stochastic dynamics to account for complex dynamics and non-deterministic dynamics in the human microbiome (our previous model assumed deterministic dynamics) and a multi-level model that takes into account both phylogeny and broader relationships that can occur with functional/metabolic similarities between distantly related species (our original model had one level and did not take into account phylogenetic structure).

**Fig. 2:**
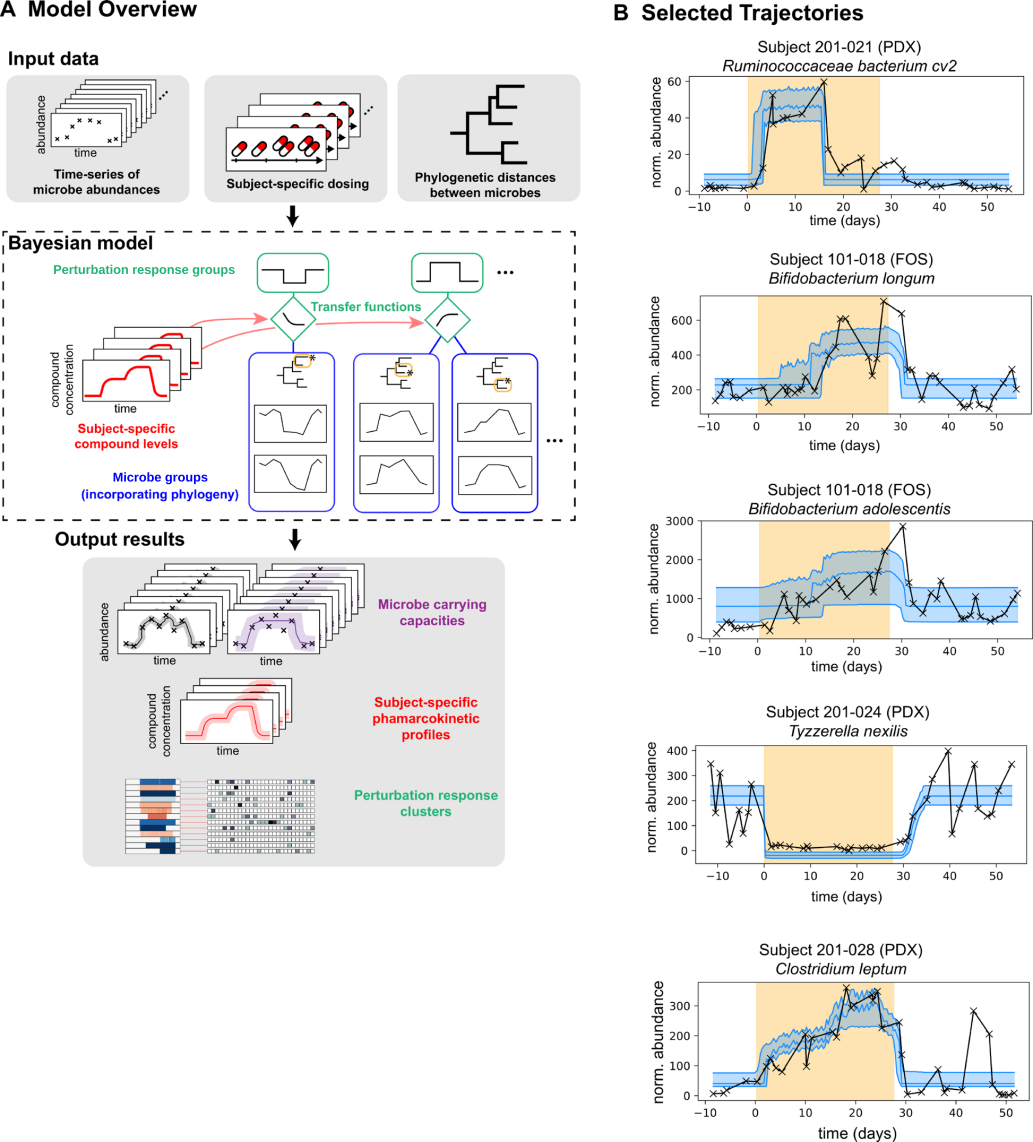
Overview of MC-TIMME2 computational model and representative individual taxa trajectories inferred from the model. **a** MC-TIMME2 is an unsupervised Bayesian nonparametric machine learning method that takes as input time-series data of microbial counts (e.g., tables of counts derived from shotgun metagenomics sequencing), dosing of the compound for each participant, and a microbial phylogenetic tree. Two levels of clustering are simultaneously learned to: (1) discover groups of common kinetic parameters (microbe groups, incorporating phylogenetic information) and common responses to the compound (perturbation groups). Microbe groups are characterized by common growth rate and carrying capacity parameters. Perturbation groups are characterized by a time-period of activity (including possible time-delays) and a magnitude of the perturbation, modulated by inferred participant-specific compound levels passed through a nonlinear transfer function. Microbe groups are super-clustered into perturbation groups. MC-TIMME2 produces several outputs, including carrying capacities (estimated steady-state levels on and off perturbations), inferred participant-specific compound concentrations over time, and maps of perturbation response clusters. **b** Trajectories of individual taxa inferred by MC-TIMME2, selected to highlight model capabilities. *Ruminococcaceae bacterium* cv2 in subject 201-021 shows a strong positive perturbation effect that ends before the end of the feeding period. *Bifidobacterium longum* and *Bifidobacterium adolescentis* in subject 101-018 demonstrate a delayed positive response that does not start until about 10 days after the start of compound administration. *Tyzzerella nexilis* in subject 201-024 shows a strong negative response. *Clostridium leptum* in subject 201-028 shows an increase in the magnitude of the response as the dose of the compound was increased

MC-TIMME2 takes as input time-series data of microbial counts (e.g., tables of counts of taxa derived from shotgun metagenomics sequencing), time-varying compound dosing data for each participant, and a bacterial phylogenetic tree (Fig. 2a) and outputs several summaries of model inferences including carrying capacities of each taxon, subject-specific pharmacokinetics of the compound, and groups of taxa exhibiting similar dynamic behaviors or responses to the compound. MC-TIMME2 is an unsupervised machine learning method, which automatically learns two levels of grouping using Bayesian nonparametric techniques: at the bottom level, groups of microbes (microbe group) with similar phylogeny and kinetic behaviors are learned, which are then further grouped at the next level (super-groups or perturbation groups) to account for similar responses to a given perturbation (i.e., feeding of the compound in this case). Each perturbation group encodes kinetic response parameters, including the time-window of response (including potential time-delays from feeding) characterized by an onset time and duration. In addition, each perturbation group encodes the magnitude of the perturbation effect specific to each participant, which takes into account a participant-specific pharmacokinetic profile. Thus, all taxa in a perturbation group share a common time window of activity and a common dose-response relationship to the compound. Because multiple microbe groups can belong to the same perturbation group, MC-TIMME2 can discover phylogenetically heterogeneous taxa that exhibit similar kinetic responses to a perturbation. MC-TIMME2 is fully Bayesian, so results consist of posterior distributions over parameters. An advantage of our Bayesian nonparametric approach is that inferences for individual taxa can also “borrow strength” from other taxa and thus more robustly estimate parameters for the individual taxa [18]; see Fig. 2b for selected examples of individual taxa trajectories. To characterize confidence in inferences, we exploit standard Bayesian model interpretations, including Bayes factors. See the “Methods” section and Supplemental Methods for complete details of the MC-TIMME2 model and inference algorithm.

### MC-TIMME2 analyses reveal differing kinetics of individual taxa in the gut microbiome responses to FOS and PDX

MC-TIMME2 allows us to quantitate kinetic parameters of the response to the compounds, namely the *onset time* and *duration* of responses. In general, we found that responses to both compounds were relatively rapid. For those species that responded across ≥ 25% of participants (Bayes factor > 100), we found a median onset time of 1.49 days for FOS (25th percentile, 0.39 days; 75th percentile, 4.90 days) and 1.29 days for PDX (25th percentile, 0.43 days; 75th percentile, 1.93 days). The distributions of onset times for each compound are shown in Additional file 1: Figure S7. Similarly, responses to both compounds were generally sustained throughout the feeding period. We found that 83% of the species that responded across ≥ 25% of participants (Bayes factor > 100) had a perturbation duration lasting at least 70% of the feeding period for FOS and 88% for PDX.

**Fig. 3:**
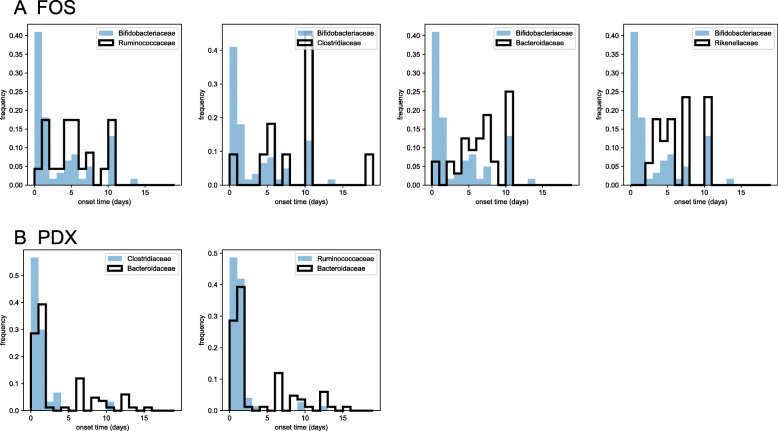
Pairs of bacterial families exhibiting significant differences in onset time of perturbation effects in response to the same compound. **a** FOS and **b** PDX. All pairs of bacterial Families that achieved significance are visualized (Mann-Whitney test, Benjamini-Hochberg corrected *p* < 0.05). Posterior medians were used to compare onset times

We found that some taxa differed significantly in their onset times in response to the same compound. For clarity, we analyzed taxa showing increases only, since this behavior was overall more consistent than taxa showing decreases. We examined both genus and family level behavior and found results were clearest at the family level. For FOS, we observed significantly faster onset times (Mann-Whitney test, *p* < 0.05) for *Bifidobacteriaceae* as compared pairwise to *Ruminococcaceae*, *Clostridiaceae*, *Bacteroidaceae*, and *Rikenellaceae* (Fig. 3a). For PDX, *Bacteroidaceae* onset times were significantly slower (Mann-Whitney test, *p* < 0.05) compared to *Clostridiaceae* and *Ruminococcaceae* (Fig. 3b).

### MC-TIMME2 identifies phylogenetically heterogeneous groups of microbes with similar kinetic responses to FOS and PDX

**Fig. 4:**
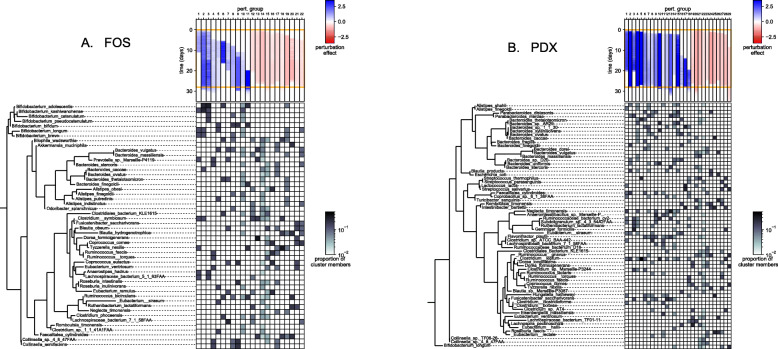
Perturbation groups inferred by MC-TIMME2 of the responses of the gut microbiome to FOS and PDX across human participants. **a** FOS and **b** PDX. Each numbered column represents a perturbation group inferred by MC-TIMME2. For each perturbation group, the top box shows the representative perturbation magnitude over time for that group, with the period of compound administration outlined in yellow. In the grid below, the species which were present in each are indicated. The colors in the grid specify the proportion of a perturbation group’s total members (with each member comprised of a time-series for a species in a participant) belonging to each species

MC-TIMME2 finds super-groups of microbes, termed perturbation groups, with similar kinetic responses; these super-groups automatically identify phylogenetically diverse groups of microbes with a similar response to the compound and provide a temporally organized, global map of such responses. For FOS (Fig. 4a), there were *Bifidobacterium* species in multiple perturbation groups, including both the earliest and latest groups of species exhibiting increases. However, the two most sustained perturbation groups (group 2 and group 3) exhibiting increases were dominated by *Bifidobacterium* species. Interestingly, these groups also contained species phylogenetically distant from *Bifidobacterium* including *Bacteroides caccae* in perturbation group 2 and *Anaerostipes hadrus* in perturbation group 3. Groups of organisms exhibiting decreases showed less clear trends along phylogenetic lines, although perturbation group 14 (a group exhibiting decreases and an early onset time) contained Clostridial species *Dialister invisus*, *Blautia obeum*, *Coprococcus comes*, and *Ruminococcus torques*. Regarding the duration of responses, we saw heterogeneity of durations, although in general this was driven by delayed onset times with most responses lasting until the end of the feeding period with the exception of perturbation groups 1, 6, and 7.

For PDX (Fig. 4b), MC-TIMME2 found more perturbation groups overall. Among groups exhibiting increases, several contained multiple *Bacteroidiales* species including *Bacteroides caccae*, *B. stercoris*, *B. vulgatus*, *B. thetaiotaomicron*, *Parabacteroides distasonis*, and *Parabacteroides merdae*. As with FOS, groups exhibiting decreases showed less clear trends along phylogenetic lines. For PDX, we observed less heterogeneity in onset time and duration than with FOS.

## Discussion

To our knowledge, our study represents the highest temporal and microbiological resolution characterization of microbiome responses to dietary glycans in human participants to date. Overall, we found consistency of responses to glycans across human participants, in terms of alterations in microbiome ecological diversity and community structure as well as changes in specific taxa, even at lower doses of compounds or in crossover studies with two compounds. Our results are consistent with prior studies that show FOS can promote *Bifidobacterium* spp. [49, 50] and PDX can promote *Bacteroides* and *Parabacteroides* species [5], but provide greater insight into additional responding species as well as the detailed kinetics of their responses.

Our purpose-built computational method, MC-TIMME2, enabled us to quantitate key kinetic properties, onset time and duration, of the response to FOS and PDX. In the case of FOS, we saw faster onset times for *Bifidobacteriaceae* as compared to *Ruminococcaceae*, *Clostridiaceae*, *Bacteroidaceae*, and *Rikenellaceae.* These results are consistent with a role of *Bifidobacteriaceae* as primary degraders of FOS, with species in the other families listed above responding later to metabolites (e.g., intermediate degradation products) or other conditioning effects of *Bifidobacteriaceae* growth in the gut. For PDX, consistent with prior studies, we found that *Bacteroidaceae* organisms were dominant responders to the compound. However, the onset times of *Bacteroidaceae* organisms were significantly slower compared to *Clostridiaceae* and *Ruminococcaceae*. These results suggest that while the response of *Clostridiaceae* and *Ruminococcaceae* species to PDX in the human gut is not as strong or as consistent as for *Bacteroidaceae* species, *Clostridiaceae* and *Ruminococcaceae* species still respond to PDX and may be able to utilize it faster. These findings have important implications for future studies and the need for dense temporal sampling experimental designs and computational methods capable of analyzing such data in detail. Endpoint studies or analyses looking only at responses of dominant taxa will miss time-varying behavior and less abundant organisms that may still be biologically relevant. Overall, the diverse response patterns of taxa may be important for evaluating glycan effects on human health. For instance, knowledge of response rate kinetics could be important in determining the timing and length of treatment to take into account both faster and slower responding taxa.

Our analyses suggest that PDX may cause diverse alterations in the metabolic potential of the microbiome beyond just the capacity to metabolize the compound itself. In previous work in a well-controlled mouse-model of microbiome perturbations [51], we showed that bacteriophages targeting single species caused widespread cascading changes in abundances of non-targeted species with concomitant effects on the gut metabolome. Our present study suggests similar cascading effects of taxa abundances and thus could explain the broad changes in carbohydrate-utilization gene abundances observed. Another possibility is that bacteria capable of metabolizing PDX, a synthetic glucose polymer with randomly cross-linked bond types, tend to have extensive carbohydrate utilization capabilities that “come along for the ride” when these organisms increase in abundance with PDX feeding. However, these results must be interpreted with caution. Our analysis method only used taxa abundances estimated from shallow shotgun metagenomic data and effectively inferred gene abundances based on mapping to reference genomes, meaning the mapping confirms the gene’s presence or absence in the reference but cannot confirm the gene’s presence or absence in strains in specific samples. Further, even if we had directly assembled genes from metagenomic data, one cannot conclude that the gene’s predicted function is active in the conditions assayed.

A related issue is false negatives in our genomic analyses of carbohydrate-active functions. For example, GH32, a β-fructosidase gene, is known to be important for metabolizing FOS. Although there was an increase in abundance for this gene during FOS feeding, the increase was relatively minor and did not rise to the level of statistical significance in our analysis. We observed that GH32 is fairly common throughout the microbiome and many taxa annotated as encoding GH32 do not increase in abundance with FOS feeding. This could be caused by the reasons discussed above, including strains lacking the gene even if it is present in the reference genomes or the enzyme not being active despite being encoded in the genome. Another possibility is that the enzyme was active in the strains in question and they metabolized FOS, but competitive interactions with other members of the microbiome drove abundances of the strains down. Given these complexities, studies that more directly assess the metabolic activity of the microbiome through methods such as metatranscriptomics and metabolomics will be important in elucidating the extent of metabolic remodeling induced by glycans.

As we have previously described, time-series analyses can also generate hypotheses about niche similarities among phylogenetically diverse bacteria (MC-TIMME [18]). In the present study, we observed co-clustering based on similar dynamics of *Bacteroides* and *Parabacteroides* species with *Fusicatenibacter saccharivorans*, a species within the family *Lachnospiraceae*. Little is known about *F. saccharivorans*, although it has previously been reported as decreased in participants with ulcerative colitis [52]; our finding that this organism shares a similar temporal response pattern to *Bacteroides* and *Parabacteroides* suggests these organisms may share functional characteristics and thus could provide insights into *F. saccharivorans* biology.

From the perspective of the design of human studies around glycan interventions, several of our findings suggest avenues for follow-up studies. For PDX, we observed that the dissimilarity between microbiome composition during washout and run-in periods decreased, indicating microbiome composition tended to regress toward pre-feeding composition within a short time span. In contrast, microbiomes in the FOS study maintained a high dissimilarity from the initial run-in period even during the subsequent washout period. This could either indicate lasting effects of FOS or effects due to shifting participant lifestyle such as diet. To assess these alternate explanations, future studies should monitor participants during a longer washout period and adjust for or control for additional potentially confounding variables.

## Conclusions

Using FOS and PDX as example compounds, we showed that glycan-based interventions can lead to rapid, sustained, and consistent microbiome responses. Our analyses leveraging MC-TIMME2, our purpose-built computational method for analyzing microbiome temporal dynamics in response to perturbations, characterized dynamic microbiome responses to glycans in unprecedented detail and identified putative microbial consortia exhibiting similar temporal behavior. Our studies thus lay the groundwork for a detailed understanding of responses of human gut microbiomes to glycans necessary for effective use of these compounds to reshape the microbiota rationally. Such capabilities would open up new avenues for the targeted modulation of microbiomes using novel glycans and predicting microbiome dynamics upon glycan exposure with the aim of improving human health and treating diseases.

## Supplementary information

**Additional file 1.** Supplemental Figures and Table.

**Additional file 2.** Supplemental Methods.

**Additional file 3.** Supplemental Table 2. (TSV 14 kb)

## Data Availability

The sequencing data generated in the studies has been submitted to SRA under BioProject accession numbers: PRJNA594610 (https://www.ncbi.nlm.nih.gov/bioproject/PRJNA594610) [53], PRJNA594393 (https://www.ncbi.nlm.nih.gov/bioproject/PRJNA594393) [54], PRJNA594613 (https://www.ncbi.nlm.nih.gov/bioproject/PRJNA594613) [55], PRJNA594620 (https://www.ncbi.nlm.nih.gov/bioproject/PRJNA594620) [56]. The MC-TIMME2 software and source code to reproduce analyses in the manuscript is publicly available under the GNU General Public License (https://github.com/gerberlab/mctimme2) [40].
