# Supplementary material for: High-resolution temporal profiling of the human gut microbiome reveals consistent and cascading alterations in response to dietary glycans
Source: Genome Med. 2020 Jul 3;12:59. doi: 10.1186/s13073-020-00758-x (PMC7386241; doi:10.1186/s13073-020-00758-x)
Supplement: Supplementary file 1 — Additional file 1. Supplemental Figures and Table. [file 13073_2020_758_MOESM1_ESM.pdf]

Supplemental Information

Figure S1: Study design. K001 and K002 are randomized, open-label, three-cohort studies. The study product is FOS in K001 and PDX in K002. Subjects were randomized on Day 0 to one of three cohorts with different feeding amounts. The study began with 2 weeks of Run-In, 2 weeks of feeding followed by 2 weeks of feeding at same or higher dose, and 4 weeks of Washout. K003 is a randomized, double-blind, two-cohort study. Subjects were randomized on Day 0 to one of two cohorts. The study began with 2 weeks of Run-In, then 2 weeks of product 1 feeding followed by 4 weeks of Washout. Then subjects received product 2 for 2 weeks followed by another 4-week Washout.

Figure S2: Study product exposure. For each subject along the feeding periods, the intended dose of product consumption is colored in cyan and the actual dose is colored in red. The cyan and red dots overlapped on most study days for most subjects, indicating a good compliance.

Figure S3: Baseline microbiome characterization. (A) Alpha (Shannon) diversity of baseline samples in each cohort of the FOS and PDX studies. (B) Community composition at the phylum level for each subject in the FOS and PDX studies. (C) Multidimentional scaling (MDS) analysis on baseline microbiome samples in the FOS and PDX studies.

Figure S4: Species differentially abundant between baseline and feeding periods in each cohort receiving FOS or PDX.

Figure S5: Microbiome responses in the FOS-PDX crossover study. (A) Percent changes in alpha (Shannon) diversity between baseline and later periods (Feeding1, Washout1, Feeding2, and Washout2) in each cohort receiving FOS or PDX. (B) Genera differentially abundant between baseline and FOS feeding periods in cohorts that either received FOS as the first or second compound. (C) Genera differentially abundant between baseline and PDX feeding periods in cohorts that either received PDX as the first or second compound.

Figure S6. Differentially abundant genes encoding CAZymes for PDX. For each CAZyme gene, we compared the difference in the distributions of fold-change (baseline versus combined feeding periods) of aggregated abundances of taxa in participants' microbiomes with the gene, and taxa without the gene (using reference genomes to make this determination.) Statistical testing was performed using the Wilcoxon rank-sum test (with *p*-values adjusted for multiple hypothesis testing using the Benjamini-Hochberg method and FDR < 0.01.) CAZymes are organized using the CAZy database structure, which contains five enzyme classes as well as carbohydrate-binding modules. Genes are sorted by *p*-values within each enzyme class.

Figure S7: Histogram of inferred onset times. Onset times for those species which responded (Bayes factor > 100) across at least 25% of participants are shown. (A) FOS and (B) PDX.

Table S1: Demographic characteristics of participants in each study.

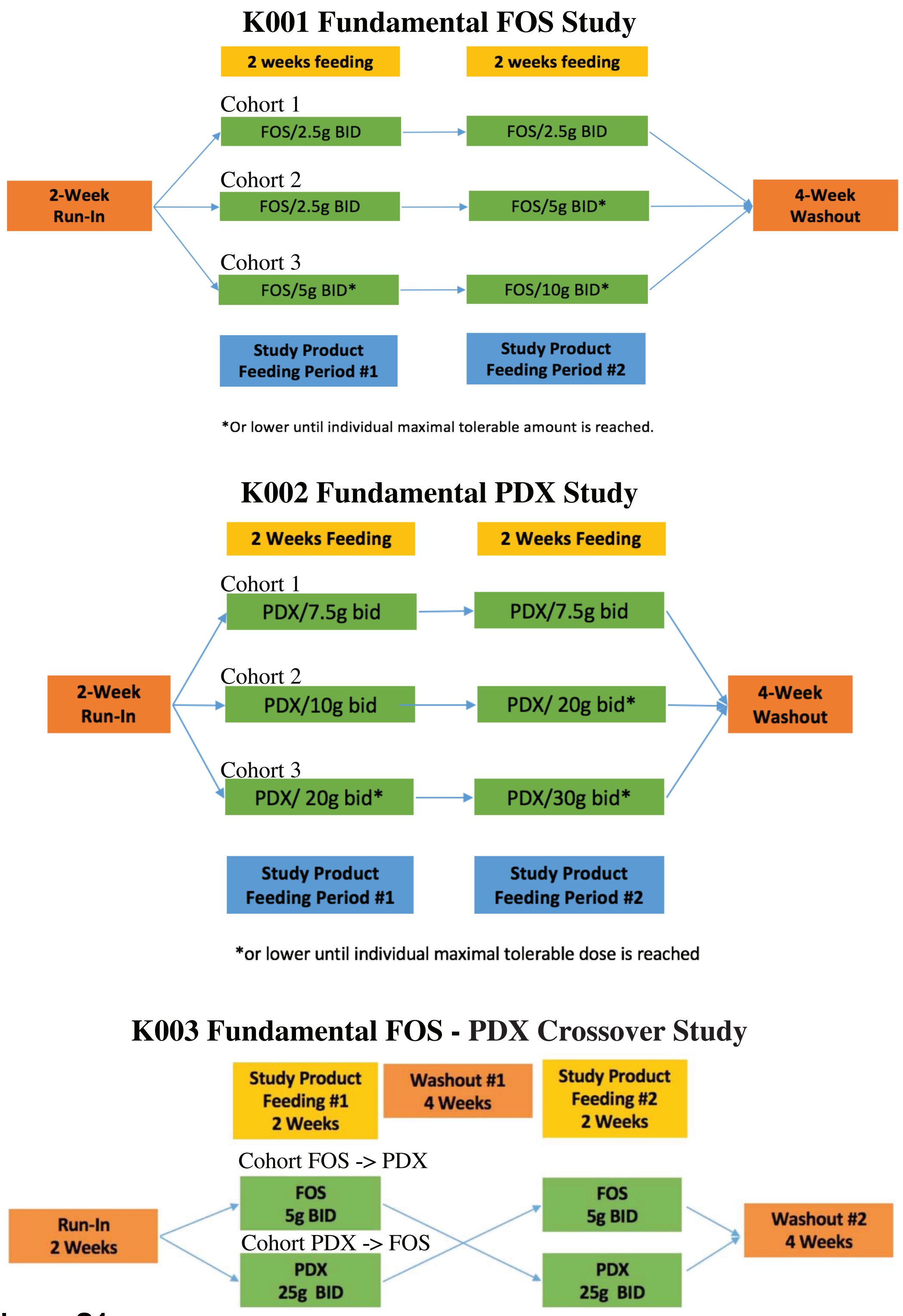
K001 Fundamental FOS Study
2 weeks feeding
2 weeks feeding
Cohort 1
FOS/2.5g BID
FOS/2.5g BID
Cohort 2
FOS/2.5g BID
FOS/5g BID*
Cohort 3
FOS/5g BID*
FOS/10g BID*
2-Week Run-In
4-Week Washout
Study Product Feeding Period #1
Study Product Feeding Period #2
*Or lower until individual maximal tolerable amount is reached.
K002 Fundamental PDX Study
2 Weeks Feeding
2 Weeks Feeding
Cohort 1
PDX/7.5g bid
PDX/7.5g bid
Cohort 2
PDX/10g bid
PDX/ 20g bid*
Cohort 3
PDX/ 20g bid*
PDX/30g bid*
2-Week Run-In
4-Week Washout
Study Product Feeding Period #1
Study Product Feeding Period #2
*or lower until individual maximal tolerable dose is reached
K003 Fundamental FOS - PDX Crossover Study
Study Product Feeding #1 2 Weeks
Washout #1 4 Weeks
Study Product Feeding #2 2 Weeks
Cohort FOS -> PDX
FOS 5g BID
Cohort PDX -> FOS
PDX 25g BID
FOS 5g BID
PDX 25g BID
Run-In 2 Weeks
Washout #2 4 Weeks

Figure S1

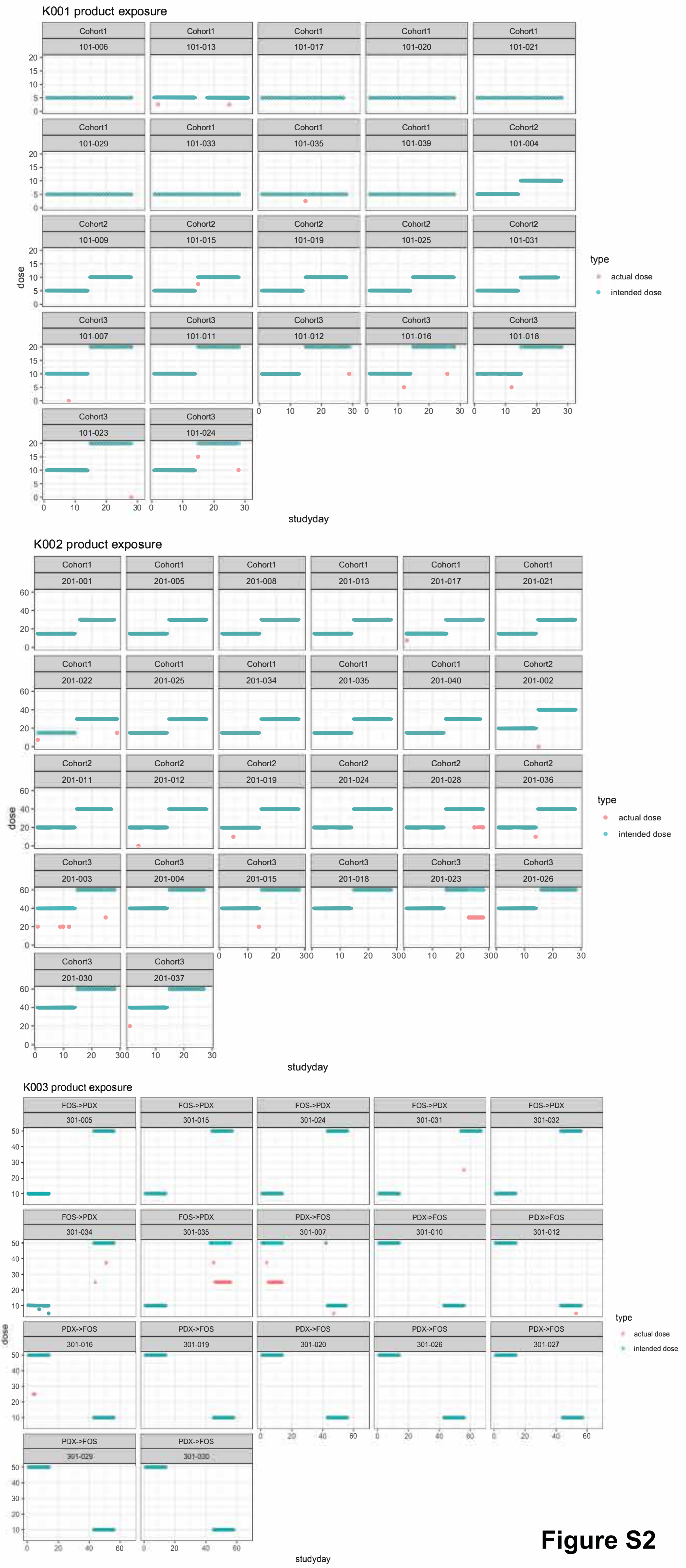
K001 product exposure
Cohort1 101-006
Cohort1 101-013
Cohort1 101-017
Cohort1 101-020
Cohort1 101-021
Cohort1 101-029
Cohort1 101-033
Cohort1 101-035
Cohort1 101-039
Cohort2 101-004
Cohort2 101-009
Cohort2 101-015
Cohort2 101-019
Cohort2 101-025
Cohort2 101-031
Cohort3 101-007
Cohort3 101-011
Cohort3 101-012
Cohort3 101-016
Cohort3 101-018
Cohort3 101-023
Cohort3 101-024
dose
studyday
type
actual dose
intended dose
K002 product exposure
Cohort1 201-001
Cohort1 201-005
Cohort1 201-008
Cohort1 201-013
Cohort1 201-017
Cohort1 201-021
Cohort1 201-022
Cohort1 201-025
Cohort1 201-034
Cohort1 201-035
Cohort1 201-040
Cohort2 201-002
Cohort2 201-011
Cohort2 201-012
Cohort2 201-019
Cohort2 201-024
Cohort2 201-028
Cohort2 201-036
Cohort3 201-003
Cohort3 201-004
Cohort3 201-015
Cohort3 201-018
Cohort3 201-023
Cohort3 201-026
Cohort3 201-030
Cohort3 201-037
dose
studyday
type
actual dose
intended dose
K003 product exposure
FOS->PDX 301-005
FOS->PDX 301-015
FOS->PDX 301-024
FOS->PDX 301-031
FOS->PDX 301-032
FOS->PDX 301-034
FOS->PDX 301-035
PDX->FOS 301-007
PDX->FOS 301-010
PDX->FOS 301-012
PDX->FOS 301-016
PDX->FOS 301-019
PDX->FOS 301-020
PDX->FOS 301-026
PDX->FOS 301-027
PDX->FOS 301-029
PDX->FOS 301-030
dose
studyday
type
actual dose
intended dose

Figure S2

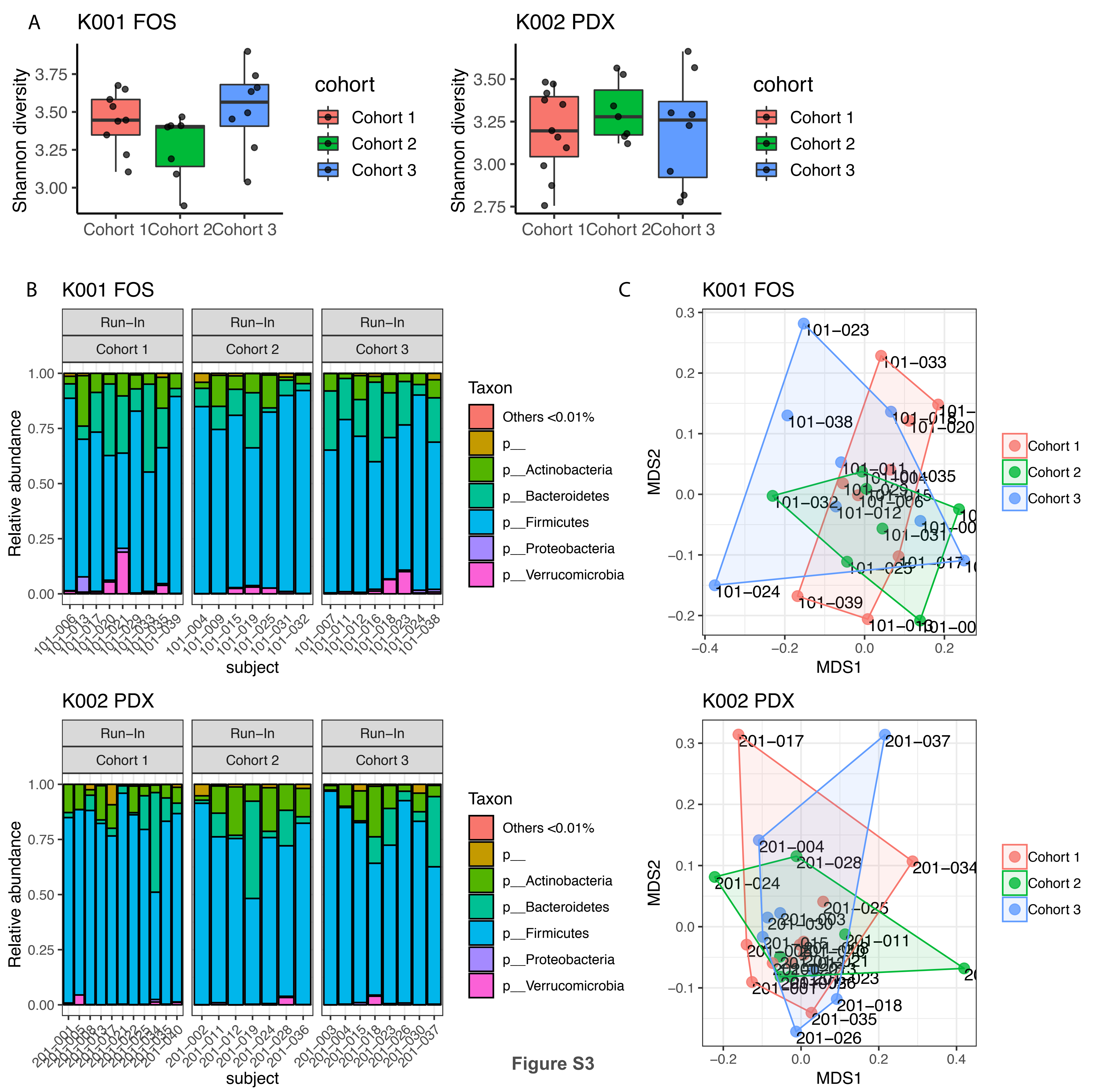
A
K001 FOS
K002 PDX
Shannon diversity
3.75
3.50
3.25
3.00
2.75
Cohort 1
Cohort 2
Cohort 3
cohort
B
K001 FOS
K002 PDX
Run−In
Relative abundance
1.00
0.75
0.50
0.25
0.00
subject
Taxon
Others <0.01%
p__
p__Actinobacteria
p__Bacteroidetes
p__Firmicutes
p__Proteobacteria
p__Verrucomicrobia
101−006
101−013
101−017
101−020
101−021
101−029
101−033
101−035
101−039
101−004
101−009
101−015
101−019
101−025
101−031
101−032
101−007
101−011
101−012
101−016
101−018
101−023
101−024
101−038
201−001
201−005
201−008
201−013
201−017
201−021
201−022
201−025
201−034
201−035
201−040
201−002
201−011
201−012
201−019
201−024
201−028
201−036
201−003
201−004
201−015
201−018
201−023
201−026
201−030
201−037
C
K001 FOS
K002 PDX
MDS1
MDS2
101−023
101−033
101−038
101−020
101−032
101−012
101−031
101−024
101−039
201−017
201−037
201−004
201−028
201−034
201−024
201−025
201−011
201−018
201−035
201−026

Figure S3

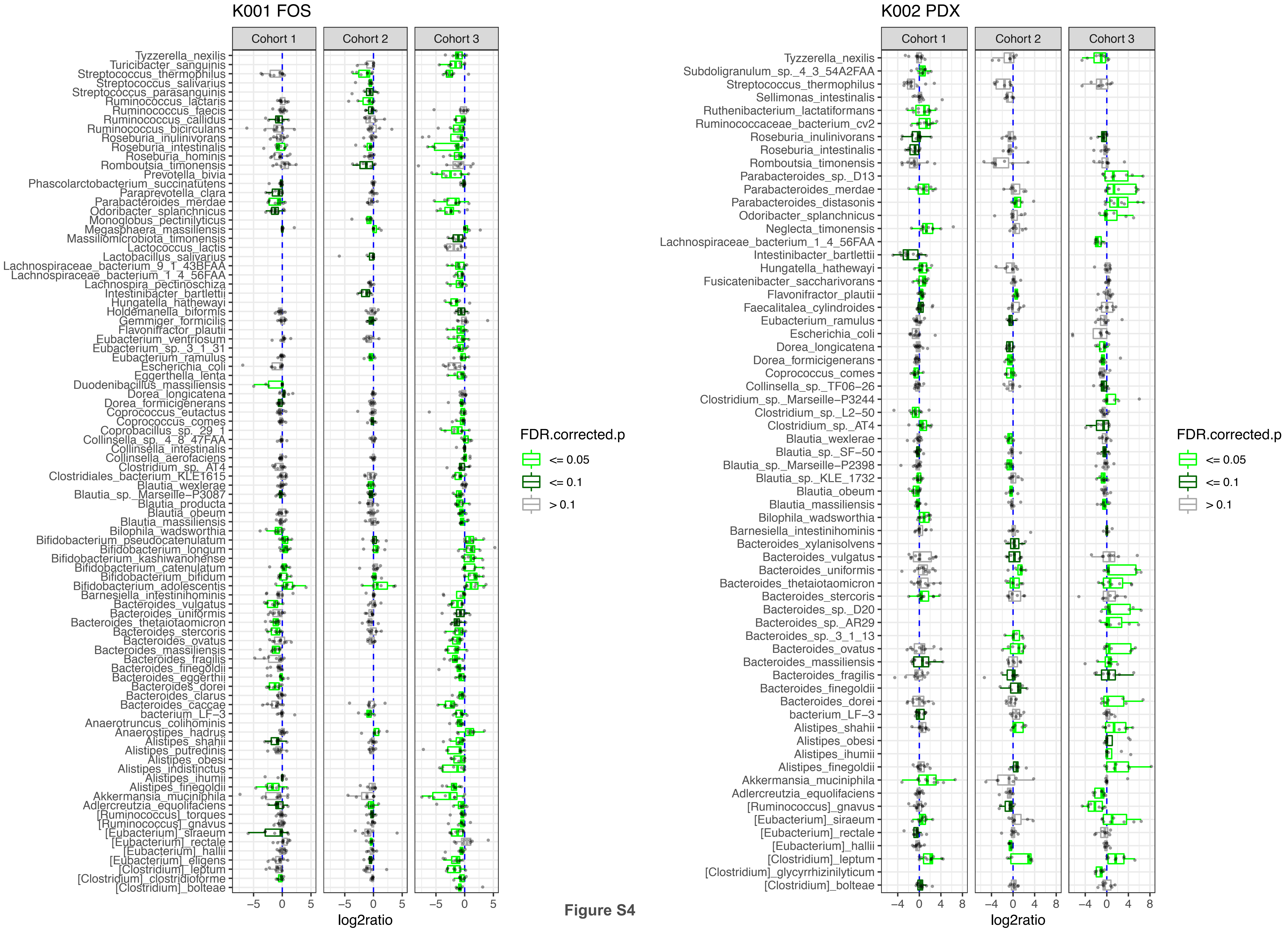
K001 FOS
Cohort 1
Cohort 2
Cohort 3
Tyzzerella_nexilis
Turicibacter_sanguinis
Streptococcus_thermophilus
Streptococcus_salivarius
Streptococcus_parasanguinis
Ruminococcus_lactaris
Ruminococcus_faecis
Ruminococcus_callidus
Ruminococcus_bicirculans
Roseburia_inulinivorans
Roseburia_intestinalis
Roseburia_hominis
Romboutsia_timonensis
Prevotella_bivia
Phascolarctobacterium_succinatutens
Paraprevotella_clara
Parabacteroides_merdae
Odoribacter_splanchnicus
Monoglobus_pectinilyticus
Megasphaera_massiliensis
Massiliomicrobiota_timonensis
Lactococcus_lactis
Lactobacillus_salivarius
Lachnospiraceae_bacterium_9_1_43BFAA
Lachnospiraceae_bacterium_1_4_56FAA
Lachnospira_pectinoschiza
Intestinibacter_bartlettii
Hungatella_hathewayi
Holdemanella_biformis
Gemmiger_formicilis
Flavonifractor_plautii
Eubacterium_ventriosum
Eubacterium_sp._3_1_31
Eubacterium_ramulus
Escherichia_coli
Eggerthella_lenta
Duodenibacillus_massiliensis
Dorea_longicatena
Dorea_formicigenerans
Coprococcus_eutactus
Coprococcus_comes
Coprobacillus_sp._29_1
Collinsella_sp._4_8_47FAA
Collinsella_intestinalis
Collinsella_aerofaciens
Clostridium_sp._AT4
Clostridiales_bacterium_KLE1615
Blautia_wexlerae
Blautia_sp._Marseille-P3087
Blautia_producta
Blautia_obeum
Blautia_massiliensis
Bilophila_wadsworthia
Bifidobacterium_pseudocatenulatum
Bifidobacterium_longum
Bifidobacterium_kashiwanohense
Bifidobacterium_catenulatum
Bifidobacterium_bifidum
Bifidobacterium_adolescentis
Barnesiella_intestinihominis
Bacteroides_vulgatus
Bacteroides_uniformis
Bacteroides_thetaiotaomicron
Bacteroides_stercoris
Bacteroides_ovatus
Bacteroides_massiliensis
Bacteroides_fragilis
Bacteroides_finegoldii
Bacteroides_eggerthii
Bacteroides_dorei
Bacteroides_clarus
Bacteroides_caccae
bacterium_LF-3
Anaerotruncus_colihominis
Anaerostipes_hadrus
Alistipes_shahii
Alistipes_putredinis
Alistipes_obesi
Alistipes_indistinctus
Alistipes_ihumii
Alistipes_finegoldii
Akkermansia_muciniphila
Adlercreutzia_equolifaciens
[Ruminococcus]_torques
[Ruminococcus]_gnavus
[Eubacterium]_siraeum
[Eubacterium]_rectale
[Eubacterium]_hallii
[Eubacterium]_eligens
[Clostridium]_leptum
[Clostridium]_clostridioforme
[Clostridium]_bolteae
-5
0
5
log2ratio
FDR.corrected.p
<= 0.05
<= 0.1
> 0.1
K002 PDX
Cohort 1
Cohort 2
Cohort 3
Tyzzerella_nexilis
Subdoligranulum_sp._4_3_54A2FAA
Streptococcus_thermophilus
Sellimonas_intestinalis
Ruthenibacterium_lactatiformans
Ruminococcaceae_bacterium_cv2
Roseburia_inulinivorans
Roseburia_intestinalis
Romboutsia_timonensis
Parabacteroides_sp._D13
Parabacteroides_merdae
Parabacteroides_distasonis
Odoribacter_splanchnicus
Neglecta_timonensis
Lachnospiraceae_bacterium_1_4_56FAA
Intestinibacter_bartlettii
Hungatella_hathewayi
Fusicatenibacter_saccharivorans
Flavonifractor_plautii
Faecalitalea_cylindroides
Eubacterium_ramulus
Escherichia_coli
Dorea_longicatena
Dorea_formicigenerans
Coprococcus_comes
Collinsella_sp._TF06-26
Clostridium_sp._Marseille-P3244
Clostridium_sp._L2-50
Clostridium_sp._AT4
Blautia_wexlerae
Blautia_sp._SF-50
Blautia_sp._Marseille-P2398
Blautia_sp._KLE_1732
Blautia_obeum
Blautia_massiliensis
Bilophila_wadsworthia
Barnesiella_intestinihominis
Bacteroides_xylanisolvens
Bacteroides_vulgatus
Bacteroides_uniformis
Bacteroides_thetaiotaomicron
Bacteroides_stercoris
Bacteroides_sp._D20
Bacteroides_sp._AR29
Bacteroides_sp._3_1_13
Bacteroides_ovatus
Bacteroides_massiliensis
Bacteroides_fragilis
Bacteroides_finegoldii
Bacteroides_dorei
bacterium_LF-3
Alistipes_shahii
Alistipes_obesi
Alistipes_ihumii
Alistipes_finegoldii
Akkermansia_muciniphila
Adlercreutzia_equolifaciens
[Ruminococcus]_gnavus
[Eubacterium]_siraeum
[Eubacterium]_rectale
[Eubacterium]_hallii
[Clostridium]_leptum
[Clostridium]_glycyrrhizinilyticum
[Clostridium]_bolteae
-4
0
4
8
log2ratio
FDR.corrected.p
<= 0.05
<= 0.1
> 0.1

Figure S4

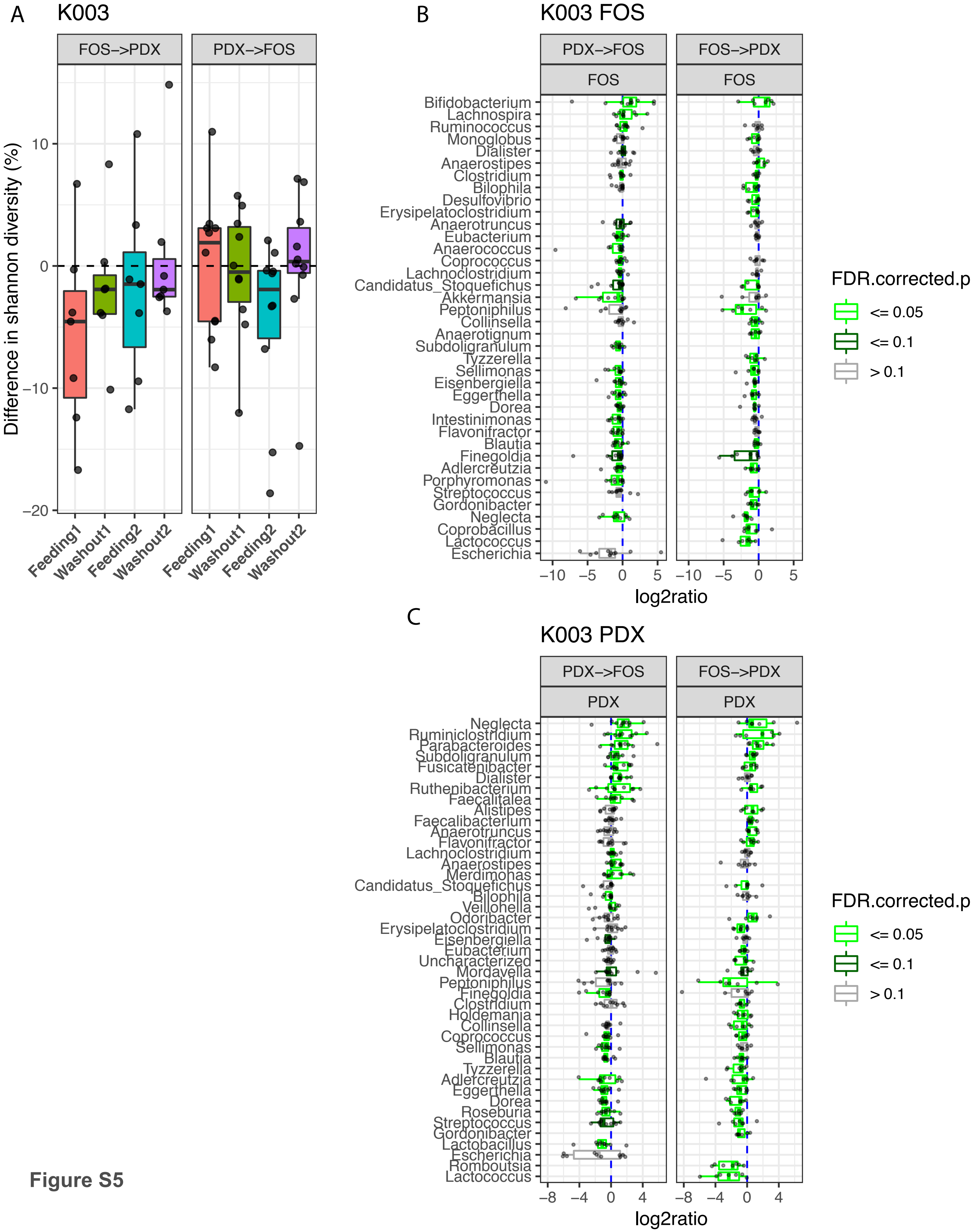
A
K003
FOS->PDX
PDX->FOS
Difference in shannon diversity (%)
10
0
−10
−20
Feeding1
Washout1
Feeding2
Washout2
B
K003 FOS
PDX->FOS
FOS->PDX
FOS
Bifidobacterium
Lachnospira
Ruminococcus
Monoglobus
Dialister
Anaerostipes
Clostridium
Bilophila
Desulfovibrio
Erysipelatoclostridium
Anaerotruncus
Eubacterium
Anaerococcus
Coprococcus
Lachnoclostridium
Candidatus_Stoquefichus
Akkermansia
Peptoniphilus
Collinsella
Anaerotignum
Subdoligranulum
Tyzzerella
Sellimonas
Eisenbergiella
Eggerthella
Dorea
Intestinimonas
Flavonifractor
Blautia
Finegoldia
Adlercreutzia
Porphyromonas
Streptococcus
Gordonibacter
Neglecta
Coprobacillus
Lactococcus
Escherichia
−10
−5
0
5
log2ratio
FDR.corrected.p
<= 0.05
<= 0.1
> 0.1
C
K003 PDX
PDX->FOS
FOS->PDX
PDX
Neglecta
Ruminiclostridium
Parabacteroides
Subdoligranulum
Fusicatenibacter
Dialister
Ruthenibacterium
Faecalitalea
Alistipes
Faecalibacterium
Anaerotruncus
Flavonifractor
Lachnoclostridium
Anaerostipes
Merdimonas
Candidatus_Stoquefichus
Bilophila
Veillonella
Odoribacter
Erysipelatoclostridium
Eisenbergiella
Eubacterium
Uncharacterized
Mordavella
Peptoniphilus
Finegoldia
Clostridium
Holdemania
Collinsella
Coprococcus
Sellimonas
Blautia
Tyzzerella
Adlercreutzia
Eggerthella
Dorea
Roseburia
Streptococcus
Gordonibacter
Lactobacillus
Escherichia
Romboutsia
Lactococcus
−8
−4
0
4
log2ratio
FDR.corrected.p
<= 0.05
<= 0.1
> 0.1

Figure S5

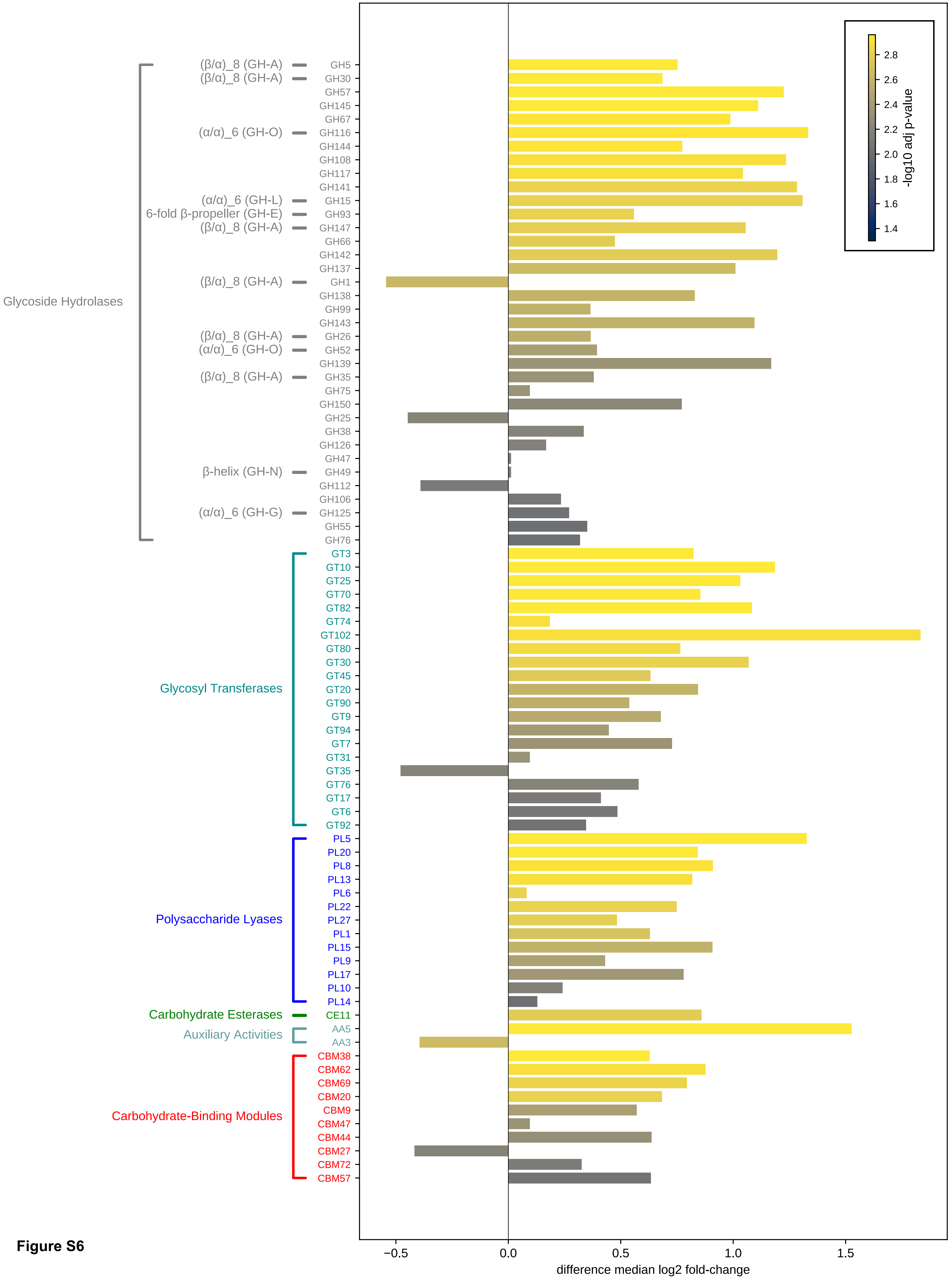
-log10 adj p-value
2.8
2.6
2.4
2.2
2.0
1.8
1.6
1.4
Glycoside Hydrolases
(β/α)_8 (GH-A)
(β/α)_8 (GH-A)
(α/α)_6 (GH-O)
(α/α)_6 (GH-L)
6-fold β-propeller (GH-E)
(β/α)_8 (GH-A)
(β/α)_8 (GH-A)
(β/α)_8 (GH-A)
(α/α)_6 (GH-O)
(β/α)_8 (GH-A)
β-helix (GH-N)
(α/α)_6 (GH-G)
GH5
GH30
GH57
GH145
GH67
GH116
GH144
GH108
GH117
GH141
GH15
GH93
GH147
GH66
GH142
GH137
GH1
GH138
GH99
GH143
GH26
GH52
GH139
GH35
GH75
GH150
GH25
GH38
GH126
GH47
GH49
GH112
GH106
GH125
GH55
GH76
Glycosyl Transferases
GT3
GT10
GT25
GT70
GT82
GT74
GT102
GT80
GT30
GT45
GT20
GT90
GT9
GT94
GT7
GT31
GT35
GT76
GT17
GT6
GT92
Polysaccharide Lyases
PL5
PL20
PL8
PL13
PL6
PL22
PL27
PL1
PL15
PL9
PL17
PL10
PL14
Carbohydrate Esterases
CE11
Auxiliary Activities
AA5
AA3
Carbohydrate-Binding Modules
CBM38
CBM62
CBM69
CBM20
CBM9
CBM47
CBM44
CBM27
CBM72
CBM57
−0.5
0.0
0.5
1.0
1.5
difference median log2 fold-change

Figure S6

A. FOS

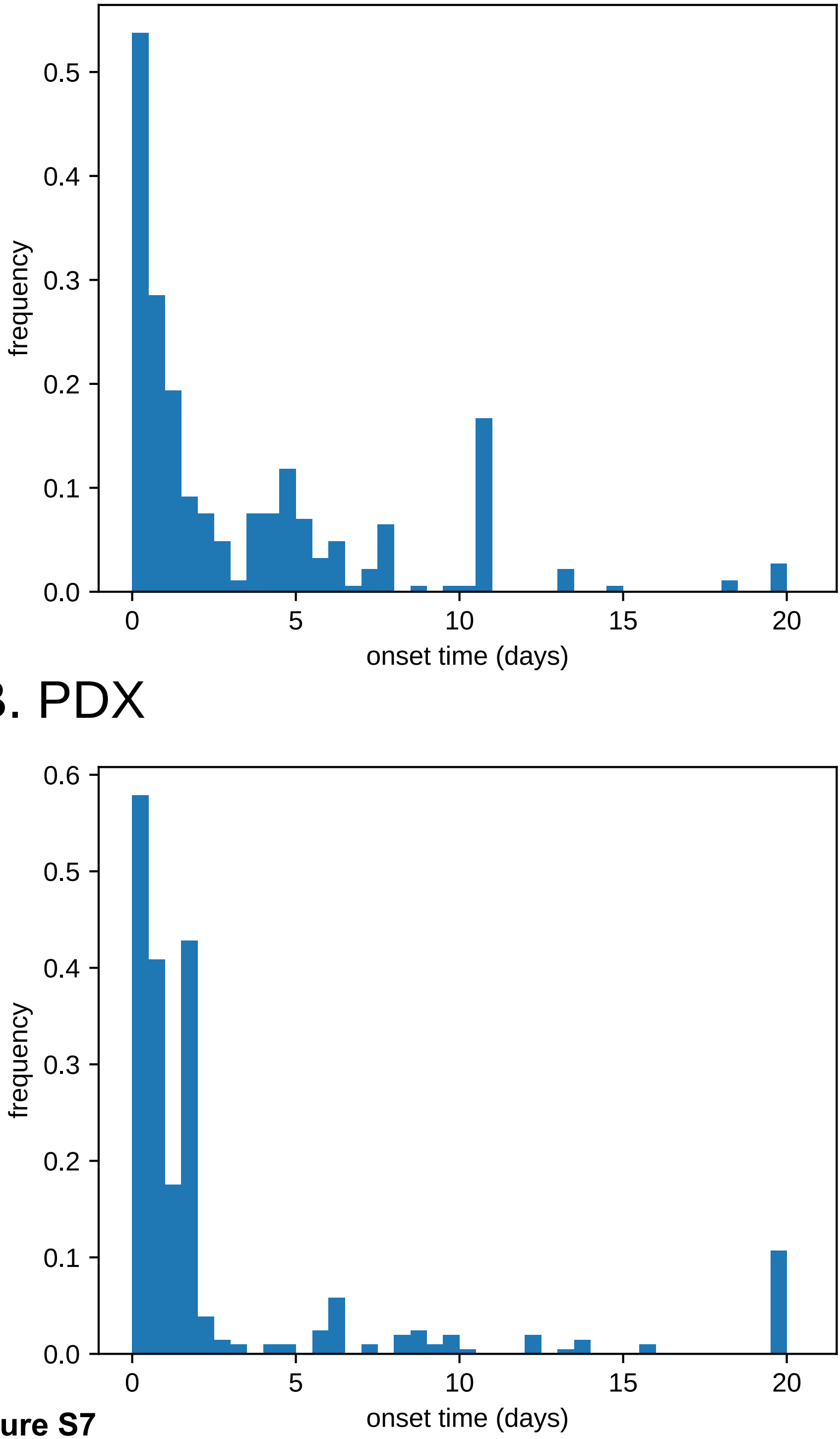

Figure S7

Table S1

K001 demographic characteristics

	Cohort1<br>n = 9	Cohort2<br>n = 6	Cohort3<br>n = 7
Gender			
Female	3 (33.3%)	4 (66.7%)	5 (71.4%)
Male	6 (66.7%)	2 (33.3%)	2 (28.6%)
Age (years)	31.9(± 11.5)	34.5(± 10.7)	38.1(± 13.1)
Race			
Asian	0 (0.0%)	1 (16.7%)	0 (0.0%)
Black or African American	0 (0.0%)	0 (0.0%)	2 (28.6%)
White	9 (100.0%)	5 (83.3%)	5 (71.4%)
Ethnicity			
Hispanic or Latino	6 (66.7%)	3 (50.0%)	4 (57.1%)
Not Hispanic or Latino	3 (33.3%)	3 (50.0%)	3 (42.9%)

K002 demographic characteristics

	Cohort1<br>n = 11	Cohort2<br>n = 7	Cohort3<br>n = 8
Gender			
Female	5 (45.5%)	4 (57.1%)	1 (12.5%)
Male	6 (54.5%)	3 (42.9%)	7 (87.5%)
Age (years)	31.9(± 10.0)	33.6(± 13.0)	31.2(± 9.6)
Race			
Asian	2 (18.2%)	1 (14.3%)	2 (25.0%)
Black or African American	0 (0.0%)	1 (14.3%)	1 (12.5%)
Native Hawaiian or Other Pacific Islander	0 (0.0%)	1 (14.3%)	0 (0.0%)
White	9 (81.8%)	4 (57.1%)	5 (62.5%)
Ethnicity			
Hispanic or Latino	5 (45.5%)	2 (28.6%)	2 (25.0%)
Not Hispanic or Latino	6 (54.5%)	5 (71.4%)	6 (75.0%)

K003 demographic characteristics

	FOS->PDX<br>n = 7	PDX->FOS<br>n = 10
Gender		
Female	6 (85.7%)	6 (60.0%)
Male	1 (14.3%)	4 (40.0%)
Age (years)	41.4(± 14.9)	34.2(± 9.9)
Race		
Black or African American	1 (14.3%)	4 (40.0%)
White	6 (85.7%)	6 (60.0%)
Ethnicity		
Hispanic or Latino	1 (14.3%)	0 (0.0%)
Not Hispanic or Latino	6 (85.7%)	10 (100.0%)
