# Supplementary material for: High-resolution temporal profiling of the human gut microbiome reveals consistent and cascading alterations in response to dietary glycans
Source: Genome Med. 2020 Jul 3;12:59. doi: 10.1186/s13073-020-00758-x (PMC7386241; doi:10.1186/s13073-020-00758-x)
Supplement: Supplementary file 2 — Additional file 2. Supplemental Methods. [file 13073_2020_758_MOESM2_ESM.pdf]

MC-TIMME2 Supplementary Methods

1 Model overview

The MC-TIMME2 model factors microbial dynamics and responses into three model components:

1. In the absence of a perturbation, each bacterial species in each subject has baseline stochastic growth dynamics
2. The concentration of the perturbing compound is assumed subject-specific, and is captured using a pharmacokinetic model
3. When a perturbation is inferred to affect a bacterial species, the perturbation modulates the baseline dynamics with a magnitude driven by the subject-specific concentration of the perturbing compound passed through a non-linear transformation

To share component 3 across microbes, we employ a double-Dirichlet Process (dDP). At the top level, the dDP forms clusters of perturbation responses, each parametrizing the non-linear transformation of the perturbing compound, as well as the time period when the perturbation is active. At the bottom level, the dDP forms clusters of bacterial trajectories, incorporating biological knowledge with a prior that encourages (but does not enforce) co-clustering of phylogenetically related species. These microbe groups are themselves assigned to perturbation clusters, allowing a single perturbation cluster to potentially capture phylogenetically distant microbes.

2 Input data and initial filtering

The input data to the model consists of sequencing counts, dosing information, and phylogenetic distances between microbes. For each subject s, we denote by $y_{so}(t_{s,i})$ the sequencing counts for OTU o at subject-specific time points $t_{s,i}$. The dosing information consists of subject-specific doses d_{si} and their times of administration a_{si}.

Before being analyzed by the model, initial filters are applied to remove bacterial time-series of no interest. An abundance filter is used to remove low-abundance species, keeping only those species which have a specified mean abundance in at least a specified number or proportion of subjects. A counts filter is then used to remove any individual time-series which does not maintain some minimum number of counts in some specified number of consecutive time points.

3 Model Details

3.1 Notation

We parametrize the negative binomial distribution using a mean m and overdispersion ϵ

$$X \sim \text{Neg-Bin}(m; \epsilon) \tag{1}$$

$$P(X = k) = \frac{\Gamma(k + 1/\epsilon)}{k!\Gamma(1/\epsilon)} \left(\frac{1/\epsilon}{1/\epsilon + m} \right)^{1/\epsilon} \left(\frac{m}{1/\epsilon + m} \right)^{k} \tag{2}$$

We parametrize the gamma distribution with a shape ω_1 and a rate ω_2

$$X \sim \text{Gamma}(\omega_1, \omega_2) \tag{3}$$

$$f_X(x) = \frac{{\omega_2}^{\omega_1}}{\Gamma(\omega_1)} x^{\omega_1 - 1} \exp(-\omega_2 x) \tag{4}$$

We parametrize the exponential distribution with a rate λ

$$X \sim \text{Exponential}(\lambda) \tag{5}$$

$$f_X(x) = \lambda e^{-\lambda x} \tag{6}$$

Normal distributions are written in terms of the variance $\text{Normal}(\mu, \sigma^2)$.

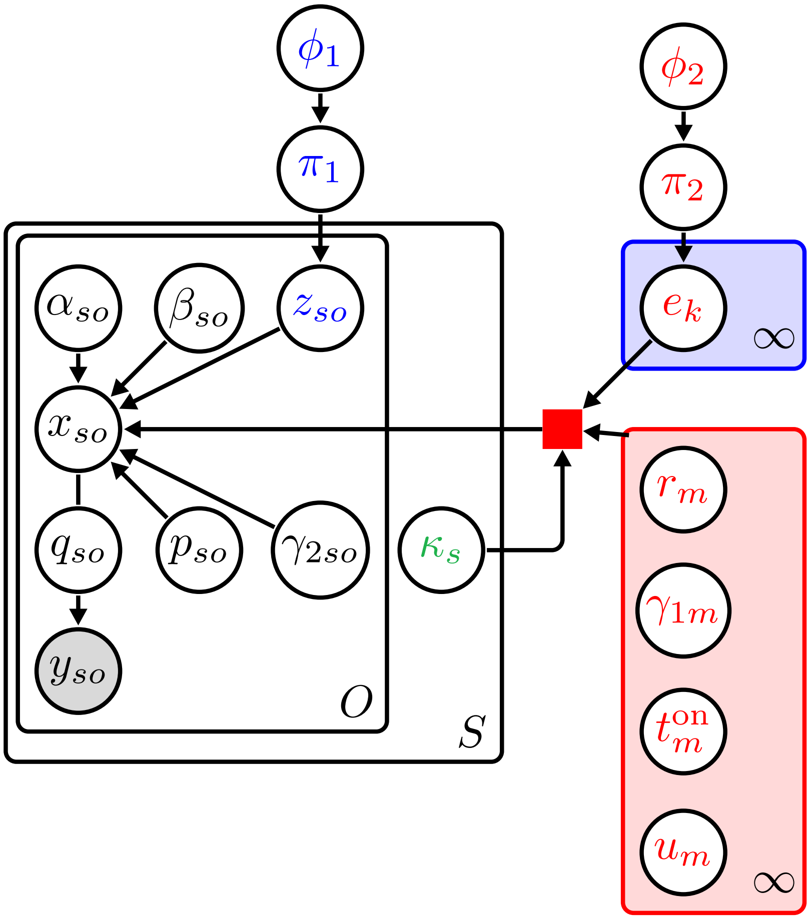

Figure 1: The MC-TIMME2 model depicted using plate notation. For brevity, some higher-level priors have been excluded. Random variables are indicated by open circles; observed data are indicated by shaded circles. Arrows indicate conditional independence relationships. Rounded rectangles indicate repeated structure. Solid squares indicate factors (functions) that link variables. The variables relevant to microbe clustering are highlighted in blue, those for perturbation clusters are highlighted in red, and the pharmacokinetics are indicated in green. ϕ_1, π_1 and z_{so} indicate the Dirichlet Process concentration parameters, mixture weights, and cluster assignments for microbe clustering, and ϕ_2, π_2 and e_k for perturbation clustering.

3.2 Dirichlet process

Our model employs Dirichlet process mixture models [1, 2]. This section includes an overview of the Dirichlet process. Mixture weights $C_1, C_2, \ldots$ are generated from a "stick-breaking" construction with concentration parameter ϕ.

$$V_1, V_2, \ldots \underset{\text{iid}}{\sim} \text{Beta}(1, \phi) \tag{7}$$

$$C_k = V_k \prod_{j=1}^{k-1} (1 - V_j) \tag{8}$$

Atom locations $\eta_1^*, \eta_2^*, \ldots$ are drawn from a base distribution G_0.

$$\eta_1^*, \eta_2^*, \ldots \underset{\text{iid}}{\sim} G_0 \tag{9}$$

Then,

$$\Theta = \sum_{k=1}^{\infty} C_k \delta_{\eta_k^*} \tag{10}$$

is a draw from a Dirichlet process with concentration parameter ϕ and base distribution G_0, or $\Theta \sim \text{DP}(\phi, G_0)$.

To specify a Dirichlet process mixture model, we introduce cluster assignment variables Z_n for each data point X_n, and a density p which takes an atom location as a parameter.

$$Z_n \sim \text{Multinomial}(C_k) \tag{11}$$

$$X_n | z_n \sim p(\cdot | \eta_{z_n}^*) \tag{12}$$

3.3 Double Dirichlet process for microbes and perturbations

In our model of the responses of the microbiota to perturbations, two levels of Dirichlet process mixture model clustering are employed. The microbe clustering Dirichlet process has a concentration parameter ϕ_1 and mixture weights π_1. Cluster assignments for each microbe o in subject s are designated by z_{so}. The microbe clusters are themselves assigned either to a null perturbation effect (with prior probability $1-\theta_{\text{perturb}}$) or to perturbation clusters in the top level of the dDP. ϕ_2 and π_2 indicate the concentration parameter and mixture weights for the perturbation DP, and e_k is the assignment to a perturbation cluster for microbe cluster k.

Phylogenetic information is incorporated into microbe clustering using a potential function ψ. Each microbe cluster chooses a representative member λ_k. Then, the likelihood for each OTU time-series being assigned to a microbe

cluster incorporates the phylogenetic distance between that OTU and the representative. Letting Ω_m indicate the perturbation parameters for perturbation cluster m,

$$P(z_{so} = k | x_{so}, e_k, \lambda_k) \propto P(z_{so} = k) P(x_{so} | \Omega_{e_k}) \psi(o, \lambda_k). \tag{13}$$

Letting $d(o_1, o_2)$ denote the patristic distance between species o_1 and o_2 on a phylogenetic tree, the potential function is given by

$$\psi(o, \lambda_k) = \exp(-\zeta_0 d(o, \lambda_k) + \zeta_1) \tag{14}$$

with hyperparameters ζ_0 and ζ_1.

3.4 Pharmacokinetics

Data for the subject-specific doses d_{si} and their times of administration a_{si} are available as inputs to the model. The level of the compound over time c in each subject s is modeled using first-order pharmacokinetics, with a subject-specific elimination rate κ_s:

$$c_s(t) = \sum_i d_{si} \exp(-\kappa_s(t - a_{si})) \mathbb{I}(t > a_{si}) \tag{15}$$

3.5 Baseline logistic growth dynamics

In the absence of a perturbation, each species is assumed to follow a stochastic logistic growth model with a growth rate α_{so} and a self-limiting term β_{so}. The latent trajectory for species o in subject s is

$$\mu_{so}(t_{s,i}) = x_{so}(t_{s,i-1}) + \alpha_{so} x_{so}(t_{s,i-1}) \Delta_{s,i} + \beta_{so}(x_{so}(t_{s,i-1}))^2 \Delta_{s,i} \tag{16}$$

$$x_{so}(t_{s,i}) \sim \text{Normal}(\mu_{so}(t_{s,i}), \Delta_{s,i} \sigma^2_{so}(t_{s,i})) \tag{17}$$

The time steps are denoted by $\Delta_{s,i} = t_{s,i} - t_{s,i-1}$. The process variance σ^2_{so} is modeled as potentially having a different value on and off the perturbation period.

$$\sigma^2_{so}(t_{s,i}) = \begin{cases} v_{so} & t_{s,i} \text{ off feeding period} \\ v^{\text{pert}}_{so} & t_{s,i} \text{ on feeding period} \end{cases} \tag{18}$$

To model the non-negativity of microbiome data, while still maintaining inference efficiency, we apply a relaxation method which has been introduced previously for microbiome dynamics [3]. Alongside the latent dynamical trajectory $x_{so}(t)$, there is a latent auxiliary trajectory $q_{so}(t)$ which is strictly non-negative (from its prior distribution) but closely coupled to $x_{so}(t)$ via a normal distribution:

$$(q_{so}(t) - x_{so}(t)) \sim \text{Normal}(0, \varphi^2_{so}(t_{s,i})) \tag{19}$$

The coupling variance φ^2 is modeled as scaling with magnitude,

$$\varphi^2_{so}(t_{s,i}) = v_1^{\text{aux}}(q_{so}(t_{s,i}))^2 + v_2^{\text{aux}} \tag{20}$$

with global parameters v_1^{aux} and v_2^{aux}.

3.6 Perturbation and persistent effect

We assume the baseline growth rate can be modulated by a perturbation. Each microbe cluster k is probabilistically assigned to either a null perturbation effect (with prior probability $1 - \theta_{\text{perturb}}$) or to a perturbation cluster e_k from the perturbation Dirichlet process. Each perturbation cluster m has a shape parameter r_m, which controls the shape of a transfer function h:

$$h(c_s(t_{s,i}); r_m) = \frac{2}{1 + \exp(-r_m c_s(t_{s,i}))} - 1 \tag{21}$$

$h(\cdot; r_m)$ reshapes the subject-specific pharmacokinetic profile c_s into a perturbation trajectory for each subject. Additionally, each perturbation cluster has a parameter γ_{1m} which sets the magnitude of the perturbation.

The period of time when a perturbation is active is given by a step function $w_{sm}(t)$ for each subject, which is parametrized by an on-time and relative duration specific to the perturbation cluster. Each perturbation cluster m has an on-time t_m^{on} given as a number of days relative to the first administration time of the compound. Additionally, the perturbation cluster m has a relative duration u_m, which specifies the perturbation duration as a proportion of each subject's full feeding period duration (the time from the first dose administration to the last administration). Assuming that $t = 0$ is the first administration time ($\min_i(a_{si}) = 0$),

$$w_{sm}(t) = \begin{cases} 1 & t_m^{\text{on}} < t < t_m^{\text{on}} + u_m \max_i(a_{si}) \\ 0 & \text{else} \end{cases} \tag{22}$$

Each time-series may optionally have a persistent effect which continues to modulate the dynamics after the compound has been excreted. The parameters for these persistent effects are specific to each OTU time-series. The prior probability for the persistent effect being active is given by θ_{persist}. An indicator variable b_{so} specifies whether or not the persistent effect is present, and γ_{2so} controls the magnitude of the persistent effect. The period when the persistent effect is active is given by a step function $p_{so}(t)$, which turns on at the last dose administration and continues for t_{so}^{persist} days.

$$p_{so}(t) = \begin{cases} 1 & \max_i(a_{si}) < t < \max_i(a_{si}) + t_{so}^{\text{persist}} \\ 0 & \text{else} \end{cases} \tag{23}$$

The modulated growth rate from the compound perturbation and the persistent effect is

$$\alpha'_{so}(t_{s,i}) = \alpha_{so}(1 + \gamma_{1m} w_{sm}(t_{s,i}) h(c_s(t_{s,i}); r_m) + b_{so}\gamma_{2so} p_{so}(t_{s,i})) \tag{24}$$

A microbe may have a persistent effect even when no perturbation cluster is selected, in which case the modulated growth rate is

$$\alpha'_{so}(t_{s,i}) = \alpha_{so}(1 + b_{so}\gamma_{2so} p_{so}(t_{s,i})) \tag{25}$$

3.7 Observation model and overdispersion parameter

The observed data are the sequencing counts $y_{so}(t_{s,i})$, which can be modeled by a negative binomial distribution [4], with the mean depending on the auxiliary trajectory $q_{so}(t)$ and the total counts for the sample.

$$y_{so}(t_{s,i}) \sim \text{Neg-Bin}(\eta_{so}(t_{s,i}), \epsilon) \tag{26}$$

$$\eta_{so}(t_{s,i}) = \nu_s(t_{s,i}) \frac{q_{so}(t_{s,i})}{B_s(t_{s,i})} \tag{27}$$

Here, ν_s denotes the total number of counts and B_s the bacterial concentration, which can be assumed constant when unknown.

We use a separate model applied to shallow shotgun replicates data to learn values for the overdispersion parameter ϵ. The replicates data consists of counts y_{io} for species o in replicate i, with each species assumed to have one latent mean relative abundance μ_o across all replicates. We model this data with a Dirichlet process infinite mixture model of negative binomial components, with each component having its own single value for the overdispersion parameter. This model is similar to that found in [5], which used a finite mixture of negative binomial components. Each species o in the replicates is assigned to a negative binomial cluster by an indicator variable z_o, so that $y_{io}|z_o \sim \text{Neg-Bin}(\eta_{io}, \epsilon_{z_o})$. The sample specific mean is $\eta_{io} = \nu_i \mu_o$, where ν_i is the total number of counts in sample i.

Inference is performed for this overdispersion mixture model using the replicates data, and a consensus clustering is obtained consisting of n mixture weights π_j^{noise} and overdispersion parameters ϵ_j (the procedure to obtain a consensus clustering from MCMC samples using agglomerative clustering is described below). To use these inferred values in the full time-series inference, we marginalize over assignments to the consensus clusters, assuming that

$$y_{so}(t_{s,i}) \sim \sum_{j=1}^{n} \pi_j^{\text{noise}} \text{Neg-Bin}(\eta_{so}(t_{s,i}), \epsilon_j) \tag{28}$$

3.8 Zero-inflation of auxiliary trajectory

At time points $t_{s,i}$ where the observed counts $y_{so}(t_{s,i})$ are zero, we allow for the possibility that the auxiliary trajectory at that time point is a structural zero ($q_{so}(t_{s,i}) = 0$) with a zero-inflation model. At each time point with zero counts, the auxiliary trajectory probabilistically chooses whether to be a structural zero (indicated by $\xi_{so}(t_{s,i}) = 0$), with prior probability given by a global parameter θ^{zero}. Therefore, the distribution of q can be written

$$q_{so}(t_{s,i}) \sim \begin{cases} \text{Normal}(x_{so}(t_{s,i}), \varphi^2_{so}(t_{s,i})) & y_{so}(t_{s,i}) \neq 0 \text{ or } \xi_{so}(t_{s,i}) = 1 \\ 0 & y_{so}(t_{s,i}) = 0 \text{ and } \xi_{so}(t_{s,i}) = 0 \end{cases} \tag{29}$$

4 Priors

For both Dirichlet process concentration parameters, we assume a Gamma prior

$$\phi_1 \sim \text{Gamma}(10^{-3}, 10^{-3}) \tag{30}$$

$$\phi_2 \sim \text{Gamma}(10^{-3}, 10^{-3}) \tag{31}$$

For both the probability of selecting any perturbation θ_{perturb} and the probability of selecting a persistent effect θ_{persist}, we assume a beta prior. In both cases, a beta prior with hyperparameters α and β can be interpreted as a prior observation of $\alpha - 1$ present effects and $\beta - 1$ null effects. For the perturbation selection, hyperparameters $(1, 50)$ are used, equivalent to an observation of about 50 microbe clusters all with no effect. For the persistent effect selection, the hyperparameters are $(1, 3000)$, equivalent to observing about 3000 OTU time-series (about the number getting past the filter) with no persistent effect.

$$\theta_{\text{perturb}} \sim \text{Beta}(1, 50) \tag{32}$$

$$\theta_{\text{persist}} \sim \text{Beta}(1, 3000) \tag{33}$$

A truncated normal distribution is used as the prior for the subject-specific elimination rates.

$$\kappa_s \sim \text{Trunc. Normal}(1, 1, 0.25, \infty) \tag{34}$$

The auxiliary trajectory has a uniform prior restricting it to positive values.

$$q_{so}(t) \sim \text{Uniform}(0.1, 10000) \tag{35}$$

The growth rate and the self-limiting terms both have normal priors.

$$\alpha_{so} \sim \text{Normal}(0.1, 100) \tag{36}$$

$$\beta_{so} \sim \text{Normal}(-0.02, 0.04) \tag{37}$$

The process variance parameters have inverse gamma priors. v_{so} and v_{so}^{pert} have:

$$v_{so} \sim \text{Inv. Gamma}(0.00001, 1) \tag{38}$$

The prior probability of the auxiliary trajectory choosing to be zero has a weak beta prior.

$$\theta^{\text{zero}} \sim \text{Beta}(1, 1) \tag{39}$$

The transfer function shape parameter r_m has an exponential prior

$$r_m \sim \text{Exponential}(5) \tag{40}$$

The perturbation cluster effect strengths and the persistent effect strengths use a normal prior.

$$\gamma_{1m} \sim \text{Normal}(0, 100) \tag{41}$$

$$\gamma_{2so} \sim \text{Normal}(0, 100) \tag{42}$$

The perturbation clusters are intended to capture sustained responses to the compound, and not short-term spikes. Therefore, truncated normal distributions are used as the priors for the on-time and duration, with the prior for the on-time requiring that perturbations appear within 20 days of the start of dosing.

$$t_m^{\text{on}} \sim \text{Trunc. Normal}(0, 20, 0, 20) \tag{43}$$

For the perturbation durations u_m, a truncated normal prior is also used, which requires that perturbations last at least 40% of the feeding period and places more weight on long perturbations.

$$u_m \sim \text{Trunc. Normal}(1.25, 0.1, 0.4, \infty) \tag{44}$$

The prior for the duration of the persistent effect is also a truncated normal distribution, which requires that persistent effects last at least 10 days.

$$t_{so}^{\text{persist}} \sim \text{Trunc. Normal}(30, 50, 10, \infty) \tag{45}$$

The hyperparameters ζ_0 and ζ_1 for the phylogenetic term in microbe clustering take fixed values which are calibrated by comparing the strength of the phylogenetic prior at different taxonomic levels to the strength of the Dirichlet process term which controls the probability of a microbe being assigned to a cluster based on the number of members in that cluster. In particular, amongst the species which pass initial filtering settings of 5e-4 mean abundance in 25% of subjects in our FOS study, the median distance between those in the same genus is 0.063 and those in the same family is 0.13. By requiring that the distance of 0.063 is equivalent to a cluster size of 5 members and the distance 0.13 is equivalent to a cluster size of 4 members, we get $\zeta_0 = 3.33$ and $\zeta_1 = 1.82$.

5 Inference

Inference for all parameters in the model is performed using a Markov Chain Monte Carlo method. The method uses Gibbs steps for variables with conjugate priors and Metropolis-Hastings (MH) steps otherwise. The overview of the main sampling loop is:

(A) For each species o and subject s:

1. Sample the latent trajectory x_{so} and the latent auxiliary trajectory q_{so}
2. Sample the logistic growth parameters α_{so} and β_{so}
3. Sample the process variance model parameters v_{so}, v_{so}^{pert}
4. Sample whether or not the persistent effect is present b_{so}, the persistent effect magnitude γ_{2so}, and the persistent effect duration t_{so}^{persist}
5. Sample the assignment to a microbe cluster z_{so}

(B) For each microbe cluster k:

6. Sample whether a perturbation effect is present, and if so, sample the assignment to a perturbation cluster e_k
7. Sample the representative member λ_k

(C) For each perturbation cluster m:

8. Sample the transfer function shape parameter r_m
9. Sample the perturbation on-time t_m^{on}
10. Sample the perturbation duration u_m
11. Sample the strength of the perturbation effect γ_{1m}

(D) For each subject s:

12. Sample the pharmacokinetic elimination rate κ_s

13. Sample the Dirichlet process concentration parameters ϕ_1 and ϕ_2

14. Sample the zero inflation prior probability θ^{zero}

15. Sample the perturbation selection prior probability θ_{perturb} and the persistent effect selection prior probability θ_{persist}

A single MCMC step for the whole model consists of one iteration of each of these steps. A total of $J = 15000$ MCMC steps are performed, with the first 7500 discarded as burnin. The value of a parameter at the jth MCMC iteration is indicated with a superscript (j). The total number of MCMC steps is indicated by J.

5.1 Latent trajectory and auxiliary trajectory

First consider the case where $y_{so}(t_{s,i}) \neq 0$. In this case, zero-inflation of $q_{so}(t_{s,i})$ is not possible at this time point.

At each such time point $t_{s,i}$, the joint posterior distribution for the latent trajectory $x_{so}(t_{s,i})$ and the latent auxiliary trajectory $q_{so}(t_{s,i})$ is likely to exhibit high correlation. Therefore, we achieve efficient sampling of these parameters using a multivariate Metropolis-Hastings step with adaptive proposal. MH steps of this type are described in [6].

In the following equations, the indices s and o are excluded for brevity. At each time point t_i, a multivariate normal jumping proposal distribution is used:

$$\begin{pmatrix} x(t_i)^{(*)} \\ q(t_i)^{(*)} \end{pmatrix} \sim \text{Normal}\left(\begin{pmatrix} x(t_i)^{(j-1)} \\ q(t_i)^{(j-1)} \end{pmatrix}, \Sigma_{\text{prop}}(t_i) \right) \tag{46}$$

The proposal covariance $\Sigma_{\text{prop}}(t_i)$ is set equal to the empirical covariance of the accepted trajectory and auxiliary trajectory samples at time point t_i, multiplied by a scalar which is tuned to adjust the acceptance rate towards 0.234, the optimal acceptance rate for multivariate MH [6]. During the first 75% of the burn-in period, $\Sigma_{\text{prop}}(t_i)$ is updated every 20 iterations based on the acceptance rate and samples of the last 200 steps, and for the rest of the burn-in period and after the end of the burn-in period it remains constant for all remaining iterations.

The acceptance probability for this jump depends on the unnormalized joint posterior for $x(t_i)$ and $q(t_i)$. The function l is the data likelihood:

$$l(y; q, \epsilon, \pi^{\text{noise}}) = \sum_j \pi_j^{\text{noise}} \text{Neg-Bin}\left(y; \nu \frac{q}{B}, \epsilon_j\right) \tag{47}$$

The unnormalized posterior conditional on all other parameters Ω is

$$\begin{aligned} p(x(t_i), q(t_i)|\Omega) \propto & \text{Unif.}(q(t_i); 0.1, 10000) \\ & \times l(y(t_i); q(t_i), \epsilon, \pi^{\text{noise}}) \\ & \times \text{Normal}(x(t_i) - q(t_i); 0, \varphi^2(t_i)) \\ & \times \text{Normal}(x(t_i); \mu(t_i), \Delta_i \sigma^2(t_i)) \\ & \times \text{Normal}(x(t_{i+1}); \mu(t_{i+1}), \Delta_{i+1} \sigma^2(t_{i+1})) \end{aligned} \tag{48}$$

Because the proposal is symmetric, the acceptance probability is calculated as the ratio

$$r^{\text{accept}} = \frac{p(x(t_i)^{(*)}, q(t_i)^{(*)}|\Omega)}{p(x(t_i)^{(j-1)}, q(t_i)^{(j-1)}|\Omega)} \tag{49}$$

The values for the trajectory and auxiliary trajectory for this step are given by

$$\begin{pmatrix} x(t_i)^{(j)} \\ q(t_i)^{(j)} \end{pmatrix} = \begin{cases} \begin{pmatrix} x(t_i)^{(*)} \\ q(t_i)^{(*)} \end{pmatrix} & \text{with probability } \min(1, r^{\text{accept}}) \\ \begin{pmatrix} x(t_i)^{(j-1)} \\ q(t_i)^{(j-1)} \end{pmatrix} & \text{otherwise} \end{cases} \tag{50}$$

At the time points where $y(t_i) = 0$, a structural zero of the auxiliary trajectory is possible. At these time points, an update step may also performed for the choice of a structural zero or not. This update is achieved using reversible jump MH step between two models [6]. Let M_0 denote the model with $q(t_i) = 0$ and M_1 denote the model with $q(t_i) \neq 0$. At each iteration, either a step is made to update the parameters within the current model or a jump is proposed to the other model. Letting J_{k,k^*} denote the probability of considering a move from model k to model k^*, we use $J_{0,0} = J_{1,1} = J_{0,1} = J_{1,0} = 0.5$.

For the move within model M_1, the same update as described above is used. For the move within model M_0, a single variable MH step with the same acceptance ratio as given in (49) is used, without the terms involving q.

In the case that the current model is M_0 and a jump is proposed to M_1, the reversible jump MH step is used. The augmenting variable u is drawn from $J(u)$, which is a truncated normal

$$u \sim \text{Trunc. Norm}(0, 1, 0.1, 10000) \tag{51}$$

Then, the parameter for model M_1 is defined by setting $q(t_i) = u$ and leaving all other parameters at the same values. The acceptance ratio for this move is

$$r = \frac{p(x|q = u, M_1)p(q = u)p(q \neq 0)}{p(x|q = 0, M_0)J(u)p(q = 0)} \tag{52}$$

With probability $min(1, r)$ the move to M_1 with the given value of $q(t_i)$ is accepted. In the case that the current model is M_1 and a jump is proposed to M_0, an augmenting variable is not required, as the proposal just sets $q(t_i) = 0$. Then the acceptance ratio is

$$r = \frac{p(x|q = 0, M_0)J(u)p(q = 0)}{p(x|q = u, M_1)p(q = u)p(q \neq 0)} \tag{53}$$

5.2 Logistic growth parameters

The growth rate α_{so} and the self-limiting term β_{so} have conjugate Normal priors, so they are sampled using straightforward Gibbs steps.

5.3 Process variance parameters

The process variance parameters have conjugate inverse Gamma priors, so they are sampled using standard Gibbs steps.

5.4 Persistent effect selection and effect magnitude

The persistent effect magnitude γ_{2so} has a conjugate Normal prior, and is updated with a Gibbs step. The indicator variable controlling the presence of the persistent effect is updated with a Gibbs step.

5.5 Microbe cluster assignments

To sample assignments in the double Dirichlet Process, we use a Gibbs sampling method similar to [7]. In the first step, we sample assignments of bacterial trajectories to microbe clusters, marginalizing over the probabilities for the perturbation clusters.

In these equations, $F_m(x_{so})$ denotes the likelihood of the trajectory x_{so} under the perturbation cluster m:

$$F_m(x_{so}) = \prod_i \text{Normal}(x_{so}(t_{s,i}); \mu_{so}^{(m)}(t_{s,i}), \Delta_{s,i}\sigma_{so}^2(t_{s,i})) \tag{54}$$

where $\mu_{so}^{(m)}$ indicates the μ computed with the perturbation parameters from perturbation cluster m:

$$\begin{aligned}\mu_{so}^{(m)}(t_{s,i}) = x_{so}(t_{s,i-1}) + (\alpha_{so}(1 + \gamma_{1m} w_{sm}(t_{s,i}) h(c_s(t_{s,i}); r_m) \\ + b_{so}\gamma_{2so} p_{so}(t_{s,i})))x_{so}(t_{s,i-1})\Delta_{s,i} + \beta_{so}(x_{so}(t_{s,i-1}))^2\Delta_{s,i}\end{aligned} \tag{55}$$

Conditional on the other parameters, the posterior distribution for the cluster assignment variable z_{so} is

$$P(z_{so} = k|\mathbf{z}^{-so}, \mathbf{e}, x_{so}) \propto \begin{cases} n_k^{-so}\psi(o,\lambda_k)F_{e_k}(x_{so}) & k \text{ exists and } n_k^{-so} > 0 \\ \phi_1\psi(o,\lambda_k = o)F_{e_k}(x_{so}) & k \text{ exists and } n_k^{-so} = 0 \\ \phi_1\psi(o,\lambda_{k^{\text{new}}} = o)p(x_{so}|z_{so} = k^{\text{new}}) & k \text{ new cluster} \end{cases} \tag{56}$$

Here, $\mathbf{z}^{-so}$ indicates all cluster assignment variables except z_{so}, and n_k^{-so} indicates the number of members in cluster k without considering time-series so. The probability for a new microbe cluster k^{new} is calculated by marginalizing over assignments to the perturbation clusters, including the null effect cluster, the existing perturbation clusters, and the possibility of a new perturbation cluster:

$$\begin{aligned}p(x_{so}|z_{so} = k^{\text{new}}) = (1 - \theta_{\text{perturb}})F_{\text{null}}(x_{so}) \\ + \theta_{\text{perturb}}\left(\sum_m \frac{n_m}{N_{\text{microbe}} + \phi_2}F_m(x_{so}) + \frac{\phi_2}{N_{\text{microbe}} + \phi_2}F_{m^{\text{new}}}(x_{so})\right)\end{aligned} \tag{57}$$

(where N_{microbe} is the total number of microbe clusters which have chosen to have a perturbation effect). Due to a lack of conjugacy for the perturbation

cluster transfer shape, on-time, and duration, the likelihood of a new perturbation cluster $F_{m^{\text{new}}}(x_{so})$ is calculated by drawing a sample from the prior for the perturbation cluster parameters (effect, transfer function shape, on-time, and duration), as in Neal's Algorithm 8 for Dirichlet Process sampling [2]. When the sample from the categorical distribution specified by (56) yields a new microbe cluster k^{new}, this new cluster is immediately assigned to a perturbation cluster according to (57):

$$P(e_{k^{\text{new}}} = m|\mathbf{e}, x_{so}) \propto \begin{cases} (1-\theta_{\text{perturb}})F_{\text{null}}(x_{so}) & m \text{ null} \\ \theta_{\text{perturb}}\frac{n_m}{N_{\text{microbe}}+\phi_2}F_m(x_{so}) & m \text{ exists} \\ \theta_{\text{perturb}}\frac{\phi_2}{N_{\text{microbe}}+\phi_2}F_{m^{\text{new}}}(x_{so}) & m \text{ new cluster} \end{cases} \tag{58}$$

5.6 Perturbation effect selection

For each microbe cluster, a Gibbs step is used to update the indicator variable for whether or not an effect is present.

5.7 Perturbation cluster assignments

In the second part of the dDP Gibbs sampler, microbe clusters are assigned to perturbation clusters or to a null effect perturbation cluster. Effect selection is sampled as described above, and microbe clusters which choose no effect are automatically assigned to the null cluster. For those microbe clusters which choose to have an effect, the posterior for the assignment to a perturbation cluster is calculated based on the likelihood of all the microbe cluster's members under each perturbation cluster.

$$P(e_k = m|\mathbf{e}^{-k}, \mathbf{x}) \propto \begin{cases} n_m^{-k} \prod_{so:z_{so}=k} F_m(x_{so}) & m \text{ exists and } n_m^{-k} > 0 \\ \phi_2 \prod_{so:z_{so}=k} F_m(x_{so}) & m \text{ exists and } n_m^{-k} = 0 \\ \phi_2 \prod_{so:z_{so}=k} F_{m^{\text{new}}}(x_{so}) & m \text{ new cluster} \end{cases} \tag{59}$$

As in sampling for the microbe clusters, the case of a new cluster is handled by drawing a sample from the prior for the transfer function shape, on-time, duration, and perturbation effect.

5.8 Microbe cluster prototypes

The microbe cluster prototype is updated using a Gibbs step. Conditional on the cluster assignments, the posterior for the prototype member λ_k is given by

$$p(\lambda_k|\mathbf{z}) \propto \prod_{so:z_{so}=k} \psi(o, \lambda_k) \tag{60}$$

5.9 Transfer function shape parameter

The transfer function shape parameters r_m are updated with MH steps. The proposal is

$$r_m^{(*)} \sim \text{Normal}(r_m^{(j-1)}, 0.01) \tag{61}$$

The acceptance ratio is

$$r^{\text{accept}} = \frac{\text{Expo}(r_m^{(*)}; 5) \prod_{so:e_{z_{so}}=m} F_m(x_{so}|r_m^{(*)})}{\text{Expo}(r_m^{(j-1)}; 5) \prod_{so:e_{z_{so}}=m} F_m(x_{so}|r_m^{(j-1)})} \tag{62}$$

5.10 Perturbation on-time

The perturbation on-times t_m^{on} are updated with MH steps. The proposal is

$$t_m^{\text{on}\,(*)} \sim \text{Normal}(t_m^{\text{on}\,(j-1)}, 9) \tag{63}$$

The acceptance ratio is

$$r^{\text{accept}} = \frac{\text{Normal}_+(t_m^{\text{on}\,(*)}; 0, 25) \prod_{so:e_{z_{so}}=m} F_m(x_{so}|t_m^{\text{on}\,(*)})}{\text{Normal}_+(t_m^{\text{on}\,(j-1)}; 0, 25) \prod_{so:e_{z_{so}}=m} F_m(x_{so}|t_m^{\text{on}\,(j-1)})} \tag{64}$$

5.11 Perturbation duration

The perturbation duration parameters u_m are updated with MH steps. The proposal is

$$u_m^{(*)} \sim \text{Normal}(u_m^{(j-1)}, 0.25) \tag{65}$$

The acceptance ratio is

$$r^{\text{accept}} = \frac{\text{Trunc. Normal}(u_m^{(*)}; 1.25, 0.1, 0.4, \infty) \prod_{so:e_{z_{so}}=m} F_m(x_{so}|u_m^{(*)})}{\text{Trunc. Normal}(u_m^{(j-1)}; 1.25, 0.1, 0.4, \infty) \prod_{so:e_{z_{so}}=m} F_m(x_{so}|u_m^{(j-1)})} \tag{66}$$

5.12 Perturbation effect

The perturbation effects have a conjugate prior, and are updated with Gibbs steps.

5.13 Pharmacokinetic elimination rate

The pharmacokinetic elimination rates κ_s are updated using MH steps. A normal jumping proposal is used.

$$\kappa_s^{(*)} \sim \text{Normal}(\kappa_s^{(j-1)}, \sigma_{\kappa_s}^2) \tag{67}$$

The proposal variances $\sigma^2_{\kappa_s}$ are adjusted to tune the acceptance rate towards 0.234. Every 200 iterations during the first 75% of the burn-in period, the proposal variances are tuned based on the acceptance rate of the previous 200 steps for κ_s.

The acceptance ratio for the proposal $\kappa_s^{(*)}$ is

$$r^{\text{accept}} = \frac{\text{Trunc. Normal}(\kappa_s^{(*)}; 1, 1, 0.25, \infty) \prod_o \prod_i \text{Normal}(x_{so}(t_{s,i}); \mu_{so}^{(*)}(t_{s,i}), \Delta_{s,i}\sigma^2_{so}(t_{s,i}))}{\text{Trunc. Normal}(\kappa_s^{(j-1)}; 1, 1, 0.25, \infty) \prod_o \prod_i \text{Normal}(x_{so}(t_{s,i}); \mu_{so}^{(j-1)}(t_{s,i}), \Delta_{s,i}\sigma^2_{so}(t_{s,i}))} \tag{68}$$

5.14 Dirichlet process concentration parameters

The concentration parameters for each level of the Dirichlet process are sampled according to the auxiliary variable method described in [8].

5.15 Selection prior probabilities

Both θ_{perturb} and θ_{persist} have a conjugate Beta prior and are updated by standard Gibbs steps.

6 Analysis of MCMC samples

6.1 Convergence of MCMC

We assess convergence of the MCMC chain using the $\hat{R}$ diagnostic described in [6].

6.2 Bayes factors

For each time-series, a Bayes factor for whether or not a perturbation was present is computed. The Bayes factor is the ratio of the posterior odds to the prior odds:

$$\text{BF}_{so} = \frac{p(e_{z_{so}} \neq \text{null}|D)}{p(e_{z_{so}} = \text{null}|D)} \frac{p(e_{z_{so}} = \text{null})}{p(e_{z_{so}} \neq \text{null})} \tag{69}$$

$$= \frac{\sum_j e_{z_{so}}^{(j)} \neq \text{null}}{\sum_j e_{z_{so}}^{(j)} = \text{null}} \frac{\theta_{\text{perturb},2}}{\theta_{\text{perturb},1}} \tag{70}$$

where $\theta_{\text{perturb},1}$ and $\theta_{\text{perturb},2}$ indicate the two hyperparameters of the beta prior on θ_{perturb}.

References

[1] C. E. Rasmussen, "The infinite Gaussian mixture model," in *Neural Information Processing Systems*, pp. 554–560, 2000.

[2] R. M. Neal, "Markov chain sampling methods for Dirichlet process mixture models," *Journal of Computational and Graphical Statistics*, vol. 9, no. 2, pp. 249–265, 2000.

[3] T. E. Gibson and G. K. Gerber, "Robust and scalable models of microbiome dynamics," in *International Conference on Machine Learning*, pp. 1758–1767, 2018.

[4] P. J. McMurdie and S. Holmes, "Waste not, want not: why rarefying microbiome data is inadmissible," *PLOS Computational Biology*, vol. 10, no. 4, p. e1003531, 2014.

[5] E. Bonafede, F. Picard, S. Robin, and C. Viroli, "Modeling overdispersion heterogeneity in differential expression analysis using mixtures," *Biometrics*, vol. 72, no. 3, pp. 804–814, 2016.

[6] A. Gelman, H. S. Stern, J. B. Carlin, D. B. Dunson, A. Vehtari, and D. B. Rubin, *Bayesian Data Analysis*. Chapman and Hall/CRC, 2013.

[7] A. Tayal, P. Poupart, and Y. Li, "Hierarchical double Dirichlet process mixture of Gaussian processes," in *Twenty-Sixth AAAI Conference on Artificial Intelligence*, 2012.

[8] M. D. Escobar and M. West, "Bayesian density estimation and inference using mixtures," *Journal of the American Statistical Association*, vol. 90, no. 430, pp. 577–588, 1995.
